# Rice QTL hotspots related with seed grain size, shape, weight, and color based on genome wide association study and linkage mapping

**DOI:** 10.1038/s41598-025-05814-3

**Published:** 2025-07-01

**Authors:** Rizky Dwi Satrio, Miftahul Huda Fendiyanto, Nisa Nurrahmah, Nabila Anofri, Mohammad Ikhsan, Satya Nugroho, Aris Hairmansis, M. Miftahudin

**Affiliations:** 1 Department of Biology, Faculty of Military Mathematics and Natural Science, The Republic of Indonesia Defense University, Komplek Indonesia Peace and Security Center (IPSC) Sentul, Bogor, 16810 Indonesia; 2 Department of Biology, Faculty of Mathematics and Natural Science, IPB University, Kampus IPB Dramaga, Bogor, 16680 Indonesia; 3 Research Center of Genetic Engineering, Research Organization of Life Sciences and Environment, National Research and Innovation Agency, Science Center Jl, Raya Jakarta-Bogor No.KM 46, Cibinong, Bogor, 16911 Indonesia; 4 Research Center of Food Crops, Research Organization of Agriculture and Food, National Research and Innovation Agency, Jl. Raya 9 Sukamandi, Subang, 41256 Indonesia; 5 Biomedical Engineering, Department of Electrical Engineering, Faculty of Engineering, Universitas Indonesia, Jakarta, Indonesia; 6 Research Center for Biomedical Engineering, Faculty of Engineering, Universitas Indonesia, Jakarta, Indonesia

**Keywords:** Genome-wide association study (GWAS), Grain, Hulled seed, Quantitative trait loci (QTL), Rice, Agricultural genetics, Functional genomics, Genetic linkage study, Genetic markers, Genome, Genomics, Genotype, Heritable quantitative trait, Plant genetics, Sequencing

## Abstract

Rice is a staple crop worldwide, with seed traits such as size, shape, weight, and color playing crucial roles in agricultural productivity and consumer preferences. Despite significant progress, the genetic basis underlying the variation in hulled and unhulled seed grain traits remains partially unexplored. This study presents a comprehensive analysis combining GWAS and QTL mapping to dissect the genetic architecture of hulled and unhulled seed characteristics in rice. The aim is to identify quantitative trait loci (QTLs) associated with these traits using an integration of multi-model approach genome-wide association studies (GWAS) and linkage mapping analysis. The study analyzed 244 local rice varieties for GWAS and 90 Recombinant Inbred Lines for linkage mapping analysis. The traits observed included hulled and unhulled seed grain area, perimeter, length, width, length-to-width ratio, circularity, weight, and color (RGB, HSV, Lab, yCbCr). A total of 23 QTL regions were identified, with two major QTL hotspots located on chromosomes 7 and 2. Specifically, QTL hotspots on chromosome 7 were associated with grain size, shape, and weight, while those on chromosome 2 were linked to seed color. A total of 530 SNPs significantly associated with the traits were distributed across 12 rice chromosomes, corroborating the QTL hotspot regions. Six genes on chromosome 7 and seven genes on chromosome 2 were proposed as candidate genes regulating seed grain traits. These findings provide valuable insights into the genetic control of rice seed traits and offer potential targets for breeding programs aimed at improving rice quality and yield.

## Introduction

Rice is one of the most essential staple crops on the planet, feeding more than half of the world’s population. Rice seed size, shape, and color are essential agronomic features that have a substantial influence on yield, market price, and consumer preferences. Improving these traits through genetic techniques can help enhance rice productivity to meet growing food demand. Understanding the genetic basis of these features is critical for generating new rice varieties with increased yield and quality. In recent years, there has been a surge of interest in genetic mapping of these traits in rice^1–3^.

Genetic mapping techniques such as genome-wide association studies (GWAS) and quantitative trait loci (QTL) analyses have proven to be effective for exploring the genetics of complex characteristics in rice. These techniques have been widely employed to find the genes and genetic variations that regulate several agronomic parameters in rice^4–6^. Genetic markers such as SNPs and SSRs are employed in genetic mapping to construct a genetic map of the rice genome that may be used to locate genes that influence the characteristics of interest^7–11^. QTL analysis and GWAS offer complementary approaches for understanding complex traits. QTL mapping is advantageous for detecting loci with large effects in controlled populations but is limited by low resolution and population-specific applicability, whereas GWAS provides higher resolution and can identify natural variations in diverse populations, though it requires large sample sizes and is susceptible to confounding effects. QTL analysis is a statistical tool for identifying QTLs that contribute to the variance of a complex trait based on genetic linkage maps^12–14^, whereas GWAS is an effective method for identifying genetic variants linked with complex characteristics in a population^15,16^. Multi-models GWAS were applied in this study, including the general linear model (GLM), mixed linear model (MLM), fixed and random model circulating probability unification (FarmCPU), Bayesian-information and linkage-disequilibrium iteratively nested keyway (BLINK), and settlement of MLM under progressively exclusive relationship (SUPER)^17–19^. GLM is a simple model that does not account for population structure or kinship, making it computationally efficient but prone to false positives. MLM incorporates kinship and population structure correction, reducing false positives but often being conservative and less powerful in detecting true associations. FarmCPU improves upon MLM by iteratively controlling for false positives and improving statistical power. BLINK is an enhancement of FarmCPU that further increases efficiency and power by reducing model complexity while maintaining accuracy. SUPER refines MLM by using a subset of genetic markers to better estimate kinship, improving the detection of true associations while controlling false positives. The use of multiple GWAS models in this study ensures robust and reliable identification of genetic variants by balancing statistical power and control of false discoveries, thus providing a comprehensive understanding of the genetic basis of complex traits in rice.

Seed size, shape, weight, and color are complex traits that are quantitatively inherited^20,21^. Several genes have been identified that control seed size in rice. One of these genes is FLOURY ENDOSPERM2 (*FLO2*), which regulates rice grain size and starch quality^22^. Another gene is Grain Length 4 (*GL4*), which controls longitudinal cell elongation of the outer and inner glumes and regulates grain length in African rice^23^. CLUSTERED PRIMARY BRANCH 1 (*CPB1*) is another gene that controls panicle architecture and seed size in rice^24^. Additionally, four seed size-related genes, *GS3*, *GW2*, *qSW5/GW5*, and *GIF1*, have been cloned in rice^25,26^. These genes have been shown to interact with each other to determine seed size. For example, *GS3* and *GW2* have been shown to have opposite effects on seed size, with *GS3* promoting larger seeds and *GW2* promoting smaller seeds^27^. Other genes that have been implicated in seed size regulation in rice include *WRKY53*, ACYL-CoA-BINDING PROTEIN 2 (*OsACBP2*), and Grain Width 2 (*GW2*)^28^. These genes interact with various signalling pathways and transcription factors to regulate seed size. Overall, the genetic regulation of seed size, shape, weight, and color in rice is complex and involves multiple genomic loci and candidate genes. Further research is needed to fully understand the genetic basis of these traits and their potential applications in rice breeding and production.

In this study, we conducted a comprehensive analysis of the genetic basis of seed size, shape, and color in rice using a combination of multi-models GWAS and QTL analysis. We used a diverse population of 244 accessions, including traditional and modern varieties, and 90 recombinant inbred lines derived from the IR64 x Hawara Bunar, to identify the major genes and QTLs that control these traits in rice. We used a high-density genetic map of rice^10^ and the sequence data for the 3K rice genomes^29^, to identify candidate genes associated with seed size, shape, color, and weight.

In this paper, we dissect the loci controlling seed size, shape, weight, and color in rice through GWAS and QTL Analysis. We also review the major genes and QTLs that have been identified for these traits in rice and discuss the potential applications of this research for rice breeding and improvement. Overall, our research highlights the importance of genetic mapping, QTL analysis, and GWAS for understanding the genetic basis of seed size, shape, and color in rice and for developing new rice varieties with improved yield and quality, which can help address the global food security challenges.

## Results

### Seed grain characteristics in local rice accessions and RILs population

In our experiment, we assessed the phenotypic traits of Indonesian local rice accessions, focusing on size (area, perimeter, width, length-to-width ratio), shape (circularity, roundness), weight, and color (RGB, HSV, Lab, YCbCr) of both hulled and unhulled seeds. Table 1 illustrates the genetic variation among local rice accessions based on seed size, shape, weight, and color traits. Table 2 presents the genetic variation among the recombinant inbred lines (RILs) population based on the same phenotypic traits.

**Table 1:** Genetic variation among local rice accessions based in seed size, shape, weight, and color traits.

Character	Trait	Min	Max	Mean	SD^b^	CV^c^ (%)
Size	Area_Unhulled (mm^2^)	15.18	29.85	22.36	2.96	13.25
	Perimeter_Unhulled (mm)	16.63	35.3	22.75	2.67	11.75
	Length_Unhulled (mm)	6.24	14.79	9.19	1.22	13.27
	Width_Unhulled (mm)	2.54	4.3	3.39	0.34	9.89
	LtW_Unhulled	1.76	3.92	2.74	0.41	15.12
	Area_Hulled (mm^2^)	9.16	17.74	14.15	1.63	11.52
	Perimeter_Hulled (mm)	12.29	19.85	16.89	1.27	7.52
	Length_Hulled (mm)	4.27	8.02	6.5	0.61	9.45
	Width_Hulled (mm)	2.17	3.41	2.77	0.25	9.19
	LtW_Hulled	0.32	0.7	0.55	0.06	11.58
Shape	Circularity_Unhulled	0.2	0.52	0.35	0.05	15.7
	Roundness_Unhulled	1.54	3.7	2.38	0.36	15.2
	Circularity_Hulled	0.44	0.76	0.63	0.05	8.65
	Roundness_Hulled	0.27	0.64	0.43	0.06	14.17
Weight	Seed_1000 (g)	10	40.35	25.23	4.35	17.25
Color	RGB_mR	72.52	214.89	155.12	22.36	14.42
	RGB_mG	69.87	212.09	154.71	26.36	17.04
	RGB_mB	63.47	176.3	130.28	22.27	17.1
	HSV_mH	11.96	58.2	33.24	6.83	20.55
	HSV_mS	31.83	123.13	48	13.97	29.11
	HSV_mV	75.69	215.95	158.21	22.54	14.25
	Lab_mL	75.96	214.6	160.73	24.61	15.31
	Lab_ma	121.09	140.03	124.01	3.56	2.87
	Lab_mb	130.3	147.36	141.01	2.6	1.84
	yCbCr_my	70.35	208.69	152.06	24.49	16.1
	yCbCr_mCr	126.51	147.67	130.19	3.75	2.88
	yCbCr_mCb	108.48	126.22	115.69	2.66	2.3

*Size: length to width ratio (LtW); Color: red in RGB (R), green in RGB (G), blue in RGB (B); hue in HSV (H), saturation in HSV (S), value in HSV (V), lightness in Lab (L), red/green coordinate in Lab (A), yellow/blue coordinate in Lab (B), luma component in yCbCr (Y), blue-difference chroma components in yCbCr (B), red-difference chroma components in yCbCr (B).

^b^SD: standard deviation; ^c^CV: coefficient of variation.

**Table 2:** Genetic variation among RILs Hawara Bunar x IR64 population based in seed size, shape, weight, and color traits.

Character	Trait*	Min	Max	Mean	SD^a^	CV^b^ (%)
Size	Area_Unhulled (mm^2^)	16.31	28.83	23.91	2.42	10.11
	Perimeter_Unhulled (mm)	19.73	31.19	24.67	2.16	8.74
	Length_Unhulled (mm)	7.84	13.21	10.21	0.99	9.72
	Width_Unhulled (mm)	2.65	3.74	3.2	0.19	5.99
	LtW_Unhulled	2.46	4.34	3.22	0.33	10.3
	Area_Hulled (mm^2^)	9.75	17.85	14.22	1.73	12.2
	Perimeter_Hulled (mm)	15.62	20.7	18.06	1.04	5.76
	Length_Hulled (mm)	6.09	8.37	7.13	0.46	6.48
	Width_Hulled (mm)	2	2.91	2.52	0.21	8.27
	LtW_Hulled	2.16	3.6	2.87	0.29	10.15
Shape	Circularity_Unhulled	0.35	0.61	0.5	0.05	8.97
	Roundness_Unhulled	0.2	0.4	0.3	0.03	11.13
	Circularity_Hulled	0.44	0.66	0.55	0.04	8.18
	Roundness_Hulled	0.28	0.46	0.36	0.04	10.27
Weight	Seed_1000 (g)	7.1	30.6	22.94	4.32	18.82
Color	RGB_mR	89.19	152.78	130.6	16.82	12.88
	RGB_mG	75.27	151.19	123.47	24.59	19.92
	RGB_mB	56.87	120.18	97.74	20.65	21.13
	HSV_mH	12.54	35.39	26.66	6.28	23.55
	HSV_mS	49.78	116.06	71.16	18.21	25.6
	HSV_mV	92.14	153.91	132.06	17.19	13.02
	Lab_mL	84.19	157.27	131.43	22.86	17.39
	Lab_ma	120.96	137.15	126.63	4.47	3.53
	Lab_mb	139.11	146.72	143.28	1.39	0.97
	yCbCr_my	77.69	147.93	122.67	21.74	17.72
	yCbCr_mCr	128.95	143.18	133.67	4.06	3.04
	yCbCr_mCb	110.5	118.27	113.91	1.4	1.23

*Size: length to width ratio (LtW); Color: red in RGB (R), green in RGB (G), blue in RGB (B); hue in HSV (H), saturation in HSV (S), value in HSV (V), lightness in Lab (L), red/green coordinate in Lab (A), yellow/blue coordinate in Lab (B), luma component in yCbCr (Y), blue-difference chroma components in yCbCr (B), red-difference chroma components in yCbCr (B).

^a^SD: standard deviation; ^b^CV: coefficient of variation.

The area of unhulled seeds in the local rice accession (Table 1) ranged from 15.18 mm2 to 29.85 mm2, with a mean of 22.36 mm^2^ and a standard deviation (SD) of 2.96, indicating a coefficient of variation (CV) of 13.25%. The perimeter varied from 16.63 mm to 35.3 mm, with a mean of 22.75 mm and an SD of 2.67, resulting in a CV of 11.75%. Length and width also exhibited substantial variation, with means of 9.19 mm and 3.39 mm, respectively. The length-to-width ratio (LtW) had a mean of 2.74 and a CV of 15.12%, indicating significant variability. For hulled seeds, the area ranged from 9.16 mm^2^ to 17.74 mm^2^, with a mean of 14.15 mm2 and an SD of 1.63, leading to a CV of 11.52%. The perimeter showed a mean of 16.89 mm, with a CV of 7.52%, indicating lower variability compared to unhulled seeds. Hulled seeds also displayed smaller means in length (6.5 mm) and width (2.77 mm), with the length-to-width ratio averaging 0.55 and a CV of 11.58%. Shape traits such as circularity and roundness exhibited notable variation. Unhulled seeds had a mean circularity of 0.35 and a CV of 15.7%, whereas hulled seeds had a higher mean circularity of 0.63 with a CV of 8.65%. The roundness for unhulled seeds averaged 2.38 with a CV of 15.2%, while hulled seeds had a mean of 0.43 and a CV of 14.17%. Weight, measured as the weight of 1,000 seeds, ranged from 10 g to 40.35 g with a mean of 25.23 g and a CV of 17.25%. Color traits, evaluated in different color spaces (RGB, HSV, Lab, yCbCbr), showed significant variation, with RGB values for red (mR), green (mG), and blue (mB) channels having means of 155.12, 154.71, and 130.28, respectively, and high CVs indicating substantial color diversity. HSV color parameters also varied widely, with the hue (mH) showing a mean of 33.24 and a CV of 20.55%. Lab and yCbCr color spaces exhibited similar patterns of variation, emphasizing the genetic diversity in color traits among local rice accessions.

The area of unhulled seeds in the RILs population (Table 2) ranged from 16.31 mm2 to 28.83 mm2, with a mean of 23.91 mm2 and an SD of 2.42, yielding a CV of 10.11%. The perimeter varied from 19.73 mm to 31.19 mm, with a mean of 24.67 mm and a CV of 8.74%. The length and width showed means of 10.21 mm and 3.2 mm, respectively, with the length-to-width ratio averaging 3.22 and a CV of 10.3%. Hulled seeds displayed area measurements from 9.75 mm2 to 17.85 mm2, with a mean of 14.22 mm2 and an SD of 1.73, indicating a CV of 12.2%. The perimeter had a mean of 18.06 mm and a lower CV of 5.76%. Length and width for hulled seeds showed means of 7.13 mm and 2.52 mm, respectively, with the length-to-width ratio averaging 2.87 and a CV of 10.15%. Shape traits for unhulled seeds indicated a mean circularity of 0.5 and a CV of 8.97%, while hulled seeds had a circularity mean of 0.55 with a CV of 8.18%. The roundness in unhulled seeds averaged 0.3 with a CV of 11.13%, whereas hulled seeds had a mean roundness of 0.36 and a CV of 10.27%. Weight measurements showed a range from 7.1 g to 30.6 g, with a mean of 22.94 g and a CV of 18.82%. Color traits, evaluated in RGB, HSV, Lab, and yCbCr color spaces, demonstrated significant variation, with mean RGB values for red (mR), green (mG), and blue (mB) being 130.6, 123.47, and 97.74, respectively, and high CVs indicating substantial color diversity. HSV parameters also varied, with the hue (mH) showing a mean of 26.66 and a CV of 23.55%. Lab and yCbCr color spaces followed similar patterns, emphasizing genetic diversity in color traits among the RILs population.

This study found distinct genetic differences between local rice accessions and the RIL population (Tables 1 and 2). Local rice accessions exhibited higher mean values for unhulled seed traits such as area, perimeter, and length compared to the RILs population, indicating larger and more variable unhulled seeds. Hulled seed traits also showed higher mean values in local accessions, suggesting greater size and variability. Shape traits demonstrated more consistency across both groups, although local accessions had slightly higher mean circularity and roundness values. Weight measurements were higher and more variable in local accessions, reflecting their broader genetic background. Color traits showed substantial variation in both groups, with local accessions displaying higher means and CVs, particularly in the RGB and HSV color spaces, indicating greater color diversity. Overall, local rice accessions demonstrated more significant genetic variation in seed size, shape, weight, and color traits compared to the RILs population, highlighting the diverse genetic makeup and potential for selective breeding in local rice varieties.

In this result, multiple histograms were also used to describe the phenotypic distribution of unhulled and hulled rice seed traits for both local rice accessions (Supplementary Fig. 1) and recombinant inbred lines (RILs) population (Supplementary Fig. 2). These histograms illustrate the distribution patterns and variability of each trait, providing insights into their heritability and genetic control.

The histograms for local rice accessions showed a wide range of phenotypic variability across all traits (Supplementary Fig. 1). For size traits, such as area and perimeter, the distribution indicated a right-skewed pattern, suggesting a majority of the seeds had smaller sizes with fewer larger seeds. Width and length-to-width ratio (LtW) exhibited a more normal distribution, indicating a balanced spread around the mean values. Shape traits, including circularity and roundness, also displayed normal distribution patterns, with most observations clustering around the mean, suggesting moderate variability. Weight distribution showed a broader spread, indicating significant differences in seed weight among the accessions. Color traits across different color spaces (RGB, HSV, Lab, yCbCr) exhibited diverse patterns, with some traits showing normal distribution while others had skewed distributions, reflecting the genetic diversity in color traits among the local accessions.

The histograms for the RILs population revealed distinct phenotypic distributions compared to the local accessions (Supplementary Fig. 2). Size traits, such as area and perimeter, demonstrated more normal distribution patterns, indicating a more uniform genetic background. Width and LtW traits also followed normal distributions, similar to the local accessions. Shape traits showed less variability, with circularity and roundness distributions closely aligned with normal curves, indicating stronger genetic control. Weight distribution in the RILs population was narrower, reflecting reduced variability due to the genetic homogeneity of the inbred lines. Color traits in the RILs population exhibited normal distribution patterns across most color spaces, suggesting consistent expression of these traits and strong heritability.

The phenotypic distributions observed in both local accessions and the RILs population highlight the influence of genetic and environmental factors on seed traits. Traits with normal distribution patterns, such as width, LtW, circularity, and roundness, indicate quantitative inheritance with multiple genes contributing to the trait expression. The presence of normal distributions in the RILs population suggests strong heritability and stable genetic control over these traits. Conversely, traits with skewed distributions, such as some size and color traits, indicate potential qualitative inheritance with fewer genes having a significant impact. The broader distributions and higher variability in local accessions reflect the genetic diversity and environmental influences, whereas the narrower distributions in the RILs population emphasize the effect of controlled breeding and genetic uniformity. This results provide a comprehensive view of the phenotypic distribution of seed traits in rice. The differences between local accessions and the RILs population underscore the importance of genetic background and breeding practices in shaping phenotypic traits. The observed distributions highlight the heritability of quantitative traits and the potential for targeted breeding to optimize seed characteristics in rice.

### QTL for seed size, shape, weight, and color in RILs population

In our experiment, we conducted a composite interval mapping (CIM) analysis using the recombinant inbred lines (RILs) population, integrating genotypic data from 870 bin SNPs across a 1,980 cM linkage map with phenotypic data. This analysis aimed to identify quantitative trait loci (QTL) associated with various seed grain characteristics, specifically focusing on size (area, perimeter, width, length-to-width ratio), shape (circularity, roundness), weight, and color (RGB, HSV, Lab, yCbCr).

The QTL analysis for size traits revealed significant loci on multiple chromosomes (Table 3). Notably, the QTL q.S_AR_U-7 on chromosome 7, positioned at 13.69 cM flanked by markers S7_28252688 and S7_28215463, exhibited a high LOD value of 5.75, explaining 27.21% of the phenotypic variation for seed area trait with an additive effect of -0.53. Similarly, q.S_LN_H-4 on chromosome 4 at 114.35 cM, with markers S4_27355030 and S4_27790806, showed a LOD value of 3.27 and explained 14.55% of the variation for the seed length trait. Other notable QTLs include q.S_LN_H-7 and q.S_LN_U-7, both located on chromosome 7, with significant contributions to phenotypic variation and additive effects indicating the genetic influence on seed length in both hulled and unhulled conditions. For width traits, q.S_WD_H-3 on chromosome 3 at 61.13 cM (flanked by S3_28770035 and S3_28515163) had a LOD value of 2.91, explaining 19.45% of the variation for the width trait. The analysis also detected significant QTLs for perimeter, such as q.S_PR_U-7 on chromosome 7 at 13.69 cM, with a high LOD of 7.07 and explaining 32.24% of the phenotypic variation. Shape-related traits, including circularity and roundness, were also mapped (Table 3). QTL q.F_CI_U-7 on chromosome 7 at 14.45 cM (S7_28215463) exhibited a LOD value of 3.46, explaining 13.92% of the phenotypic variation for the seed circularity trait. The roundness trait showed significant loci such as q.F_RN_U-3 on chromosome 3 at 74.49 cM (S3_26849291 and S3_26343754), with a LOD value of 2.79 and explaining 11.58% of the variation. For seed weight (Table 3), q.W_WGT-7 on chromosome 7 at 18.19 cM (S7_27021585) was a significant QTL, with a LOD value of 3.61 and explaining 23.9% of the phenotypic variation. This highlights the genetic influence on seed weight, crucial for breeding programs aiming to improve yield. Color traits, evaluated across different color spaces, revealed significant QTLs (Table 3). For instance, q.C_HSV_S-4 on chromosome 4 at 54.11 cM (S4_20697856) showed a LOD value of 2.94, explaining 15.2% of the variation. Notably, q.C_LAB_B-2.2 on chromosome 2 at 176.95 cM (S2_8034217 and S2_7426281) exhibited a high LOD value of 5.57, explaining 35.37% of the variation, indicating significant genetic control over color traits. While the analysis successfully identified QTLs for many traits, certain expected traits, such as specific color parameters (RGB), were not detected in this QTL analysis. This absence could be attributed to the complexity of these traits and the potential influence of environmental factors or the limitations of the genetic map resolution.

**Table 3:** QTLs for seed grain characteristics detected by composite interval mapping in the RIL population.

QTL*	Chr	Position^a^ (cM)	Left marker^b^	Right marker^b^	LOD^c^	r^2^ (%)^d^	Additive effect^†^
Size							
* q.S_AR_U-7*	7	13.69	S7_28252688	S7_28215463	5.75	27.21	− 0.53
* q.S_LN_H-4*	4	114.35	S4_27355030	S4_27790806	3.27	14.55	0.39
* q.S_LN_H-7*	7	11.37	S7_28324908	S7_28324908	4.4	18.19	− 0.44
* q.S_LN_U-7*	7	13.69	S7_28252688	S7_28215463	7.1	32.35	− 0.58
* q.S_LW_U-3*	3	74.49	S3_26849291	S3_26343754	3.56	13.07	− 0.45
* q.S_LW_U-7*	7	13.69	S7_28252688	S7_28215463	5.74	15.31	− 0.49
* q.S_PR_H-4*	4	114.35	S4_27355030	S4_27790806	3.7	16.23	0.41
* q.S_PR_H-7*	7	10.55	S7_28558774	S7_28324908	4.32	18.16	− 0.44
* q.S_PR_U-7*	7	13.69	S7_28252688	S7_28215463	7.07	32.24	− 0.58
* q.S_WD_H-3*	3	61.13	S3_28770035	S3_28515163	2.91	19.45	0.46
Shape							
* q.F_CI_U-3*	3	75.46	S3_26343754	S3_26343754	2.55	12.42	0.39
* q.F_CI_U-7*	7	14.45	S7_28215463	S7_28215463	3.46	13.92	0.41
* q.F_RN_U-3*	3	74.49	S3_26849291	S3_26343754	2.79	11.58	0.41
* q.F_RN_U-7*	7	14.45	S7_28215463	S7_28215463	4.69	13.54	0.45
Weight							
* q.W_WGT-7*	7	18.19	S7_27021585	S7_27021585	3.61	23.9	− 0.49
Color							
* q.C_HSV_S-4*	4	54.11	S4_20697856	S4_20697856	2.94	15.2	0.39
* q.C_LAB_B-2.1*	2	162.1	S2_8559705	S2_8559705	4.05	23.28	0.65
* q.C_LAB_B-2.2*	2	176.95	S2_8034217	S2_7426281	5.57	35.37	− 0.80
* q.C_YRB_B-2.1*	2	162.1	S2_8559705	S2_8559705	4	22.77	− 0.64
* q.C_YRB_B-2.2*	2	176.95	S2_8034217	S2_7426281	5.74	36.26	0.81

*Size: area (AR), perimeter (PR), length (LN), width (WD), length-to-width ratio (LW); shape: circularity (CI), roundness (RN); weight (WGT); color: red in RGB (R), green in RGB (G), blue in RGB (B); hue in HSV (H), saturation in HSV (S), value in HSV (V), lightness in Lab (L), red/green coordinate in Lab (A), yellow/blue coordinate in Lab (B), luma component in yCbCr (Y), blue-difference chroma components in yCbCr (B), red-difference chroma components in yCbCr (B).

^a^Location of the peak of the genetic position harbouring QTL region in centimorgans (cM).

^b^Markers flanking the right and left bins in the rice genome.

^c^Maximum log-likelihood (LOD) score, a measure of the strength of evidence for the presence of a QTL at a particular genetic position.

^d^The r^2^ value was the percentage of the total phenotypic variance for a trait that a marker accounts for.

^†^An additive effect is indicated by a positive value, suggesting similarity to the traits of the Hawara Bunar. Conversely, a negative value suggests similarity to IR64.

**Fig. 1:**
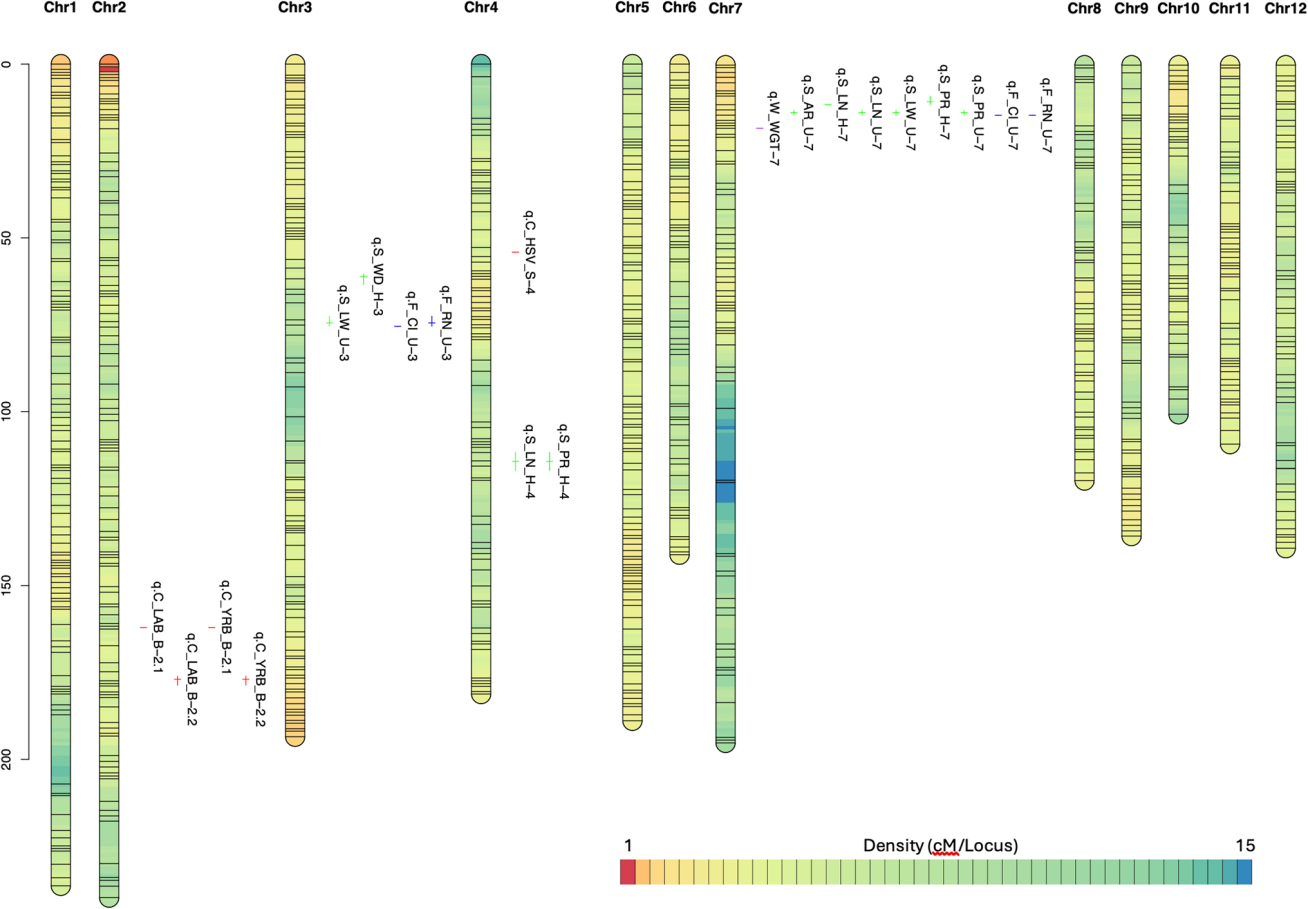
Bin linkage chromosomal map showing the locations of size (area, perimeter, length, width, length-to-width ratio), shape (circularity and roundness), weight, and color (RGB, HSV, Lab, yCbCr) for hulled and unhulled grain QTLs with the genetic distance shown in centimorgans (cM) for the rice RILs population. The color of chromosomes indicated the marker density in cM/locus.

Based on this study, we have also identified significant QTL hotspots on rice chromosomes that influence various seed-related traits (Fig. 1). A QTL hotspot is defined as a genomic region where multiple QTLs affecting different traits are co-located, indicating a region of the genome with a substantial regulatory role. Chromosomes 7 and 2 emerge as a prominent hotspot for several seed-related traits.

Chromosome 7 contains multiple QTLs related to seed size, shape, and weight, highlighting its crucial genetic influence (Fig. 1). Notably, for size traits, QTLs such as q.S_AR_U-7 (area unhulled) at 13.69 cM, q.S_LN_H-7 (length hulled) at 11.37 cM, and q.S_LN_U-7 (length unhulled) at 13.69 cM have been identified. Additionally, QTLs like q.S_LW_U-7 (length-to-width ratio unhulled) and q.S_PR_U-7 (perimeter unhulled) at the same position further emphasize this hotspot’s significance. Shape traits are also influenced by this region, with QTLs q.F_CI_U-7 (circularity unhulled) at 14.45 cM and q.F_RN_U-7 (roundness unhulled) located nearby. Furthermore, the weight trait was significantly impacted by q.W_WGT-7 (weight) at 18.19 cM. These QTLs collectively explain a substantial proportion of the phenotypic variation for their respective traits, with LOD values ranging from 2.55 to 7.10 and r2 values from 13.54% to 32.35%. The concentration of these QTLs in a relatively small chromosomal region underscores chromosome 7’s pivotal role in controlling seed size, shape, and weight traits in rice.

Chromosome 2 also presents a cluster of QTLs, particularly for color traits (Fig. 1). This chromosome contains QTLs such as q.C_LAB_B-2.1 (LAB color) and q.C_LAB_B-2.2 (LAB color) at positions 162.1 cM and 176.95 cM, respectively. Additionally, QTLs like q.C_YRB_B-2.1 (YCbCr color) and q.C_YRB_B-2.2 (YCbCr color) are located at the same positions, indicating significant genetic control over seed color in this region. These QTLs exhibit high LOD values ranging from 4.00 to 5.74 and explain phenotypic variations from 22.77% to 36.26%. The presence of multiple QTLs for color traits in this chromosomal region highlights chromosome 2 as a crucial hotspot for seed color traits.

The QTL mapping using composite interval mapping in the RIL population successfully identified significant loci associated with seed size, shape, weight, and color traits. The detected QTLs provide insights into the genetic architecture underlying these phenotypic traits, offering valuable markers for rice breeding programs aimed at enhancing desired characteristics. The high LOD values and substantial percentages of phenotypic variation explained by these QTLs underscore their importance in understanding and improving rice seed traits through genetic selection. The QTL analysis identified chromosomes 7 and 2 as key hotspots for seed-related traits in rice. Chromosome 7 harbours multiple QTLs for seed size, shape, and weight, making it a critical target for genetic studies and breeding programs. Meanwhile, chromosome 2 contains significant QTLs for color traits, providing valuable insights for improving rice seed characteristics through marker-assisted selection. These hotspots offer promising targets for further research and breeding strategies aimed at optimizing rice seed traits.

### Genomic loci associated with seed grain characteristics detected in local rice accessions

**Fig. 2:**
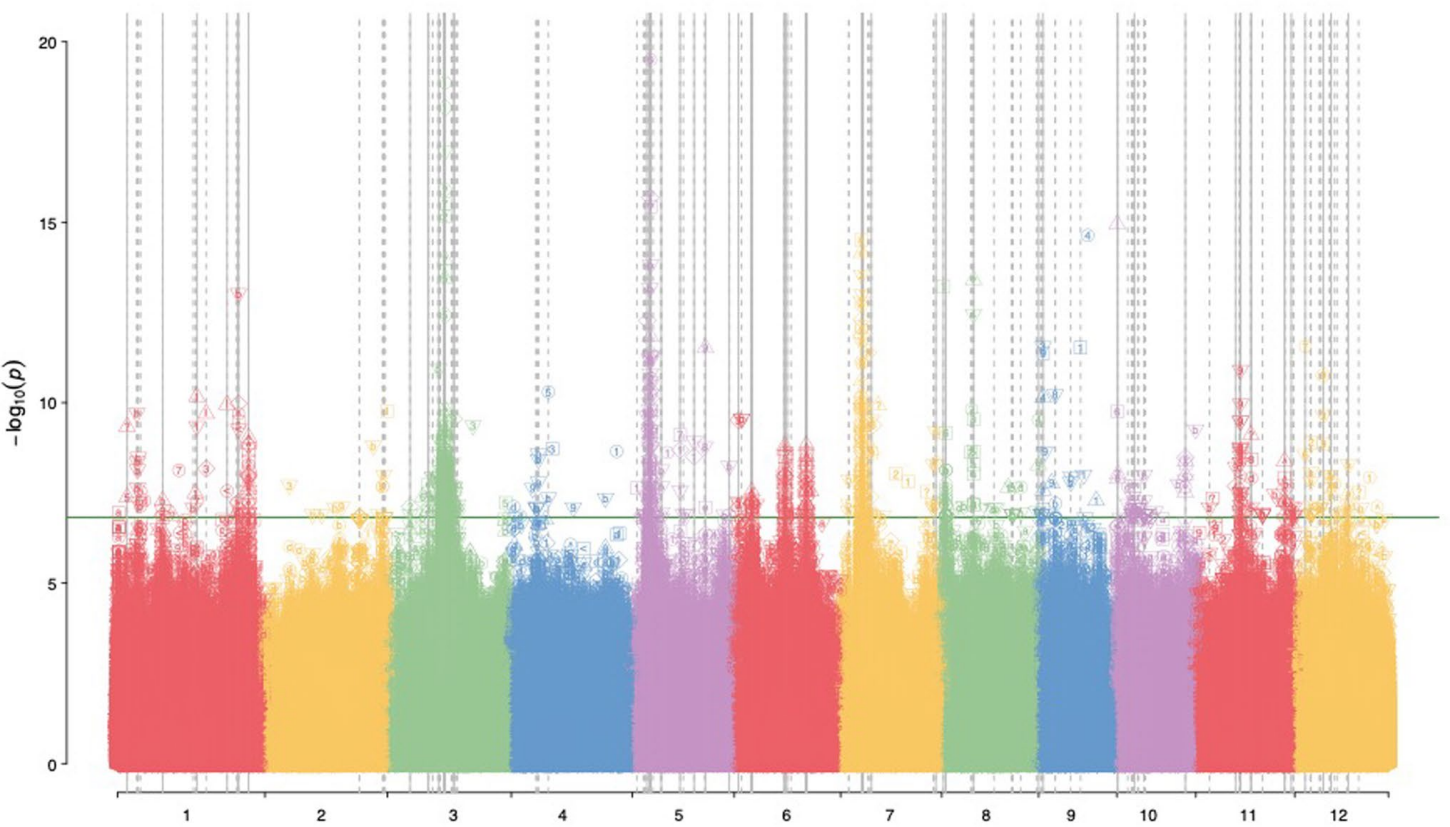
Manhattan plots based on GWAS showing the significant SNPs associated with seed grain characteristics. A total of 334,776 SNPs from 12 rice chromosomes were tested for association with seed-related traits including size (area, perimeter, length, width, length-to-width ratio), shape (circularity and roundness), weight, and color (RGB, HSV, Lab, yCbCr) for hulled and unhulled grain using five statistical models (GLM, MLM, farmCPU, BLINK and SUPER). Horizontal lines indicate the significance of the loci associated with the traits of interested. Solid vertical line indicates high consensus for loci associated with traits of interest, while a dashed line indicates imperfect consensus.

In this research, we investigated the genetic basis of various phenotypic traits in rice, specifically focusing on the hulled and unhulled seed characteristics. The phenotypic traits examined include size (area, perimeter, width, length-to-width ratio), shape (circularity, roundness), weight, and color (measured in RGB, HSV, Lab, and yCbCr color spaces). Our genetic mapping study also utilized a Genome-Wide Association Studies (GWAS) across 244 local rice accessions with a genotype dataset comprising 334,776 SNPs using multiple statistical models (GLM, MLM, farmCPU, BLINK, SUPER). Through the use of multiple statistical models to conduct GWAS, true positive SNPs associated with seed grain characteristics in rice can be detected based on their consensus results (Fig. 2). A multi-model statistical analysis approach for GWAS has also been carried out to detect consensus loci associated with quality performance in rice^30^.

Both PCA and kinship analyses revealed distinct genetic groupings among the rice accessions, reflecting the underlying population structure. The PCA conducted on the 244 local rice accessions using 334,776 SNPs revealed distinct patterns and groupings within the genetic data, forming three clusters (Supplementary Fig. 3a and 3b). The three clusters seem to represent the three major groups of rice commonly found in Indonesia, including Indica, Japonica, and Tropical Japonica, although further studies by including genotypes with known groupings still need to be done. The first principal component (PC1) explained 34.42% of the genetic variance, while the second principal component (PC2) accounted for 4.63% of the variance. The PCA scatter plot demonstrated clear clustering of the rice accessions, indicating significant genetic differentiation among them. Accessions clustering closely together were genetically similar, whereas those spread further apart exhibited greater genetic diversity. Additionally, the scree plot, showing the eigenvalues of the principal components, suggested that the majority of genetic variation could be captured by the first few components, emphasizing the effectiveness of PCA in reducing dimensionality. The kinship analysis, represented by the heatmap, illustrated the genetic relatedness among the rice accessions (Supplementary Fig. 3c). The heatmap used a color gradient to indicate the degree of relatedness, with brighter colors signifying higher genetic similarity. The hierarchical clustering, depicted by the dendrograms alongside the heatmap, further highlighted the genetic relationships among the accessions. Accessions clustered together in the dendrograms shared more genetic information, providing insights into the population structure. These groupings are crucial for understanding subpopulations within the sample and are essential for accurately conducting GWAS. By recognizing these groupings, researchers can control for population stratification and relatedness, ensuring more precise GWAS results.

Based on the provided Manhattan plots, the significant results from the GWAS for various phenotypic traits of unhulled and hulled rice seeds—such as size, shape, weight, and color—were indicated by peaks that surpass the significance threshold line in the plot. These peaks correspond to specific SNPs that show a strong association with the traits being analyzed. In the Manhattan plots, significant associations are generally distributed across multiple chromosomes, but some chromosomes show more pronounced peaks, indicating a higher concentration of significant SNPs linked to the traits. The chromosomes 1, 3, 5, 6 and 7 often display significant associations across various traits like seed size, shape, weight, and color (Fig. 2). These chromosomes are highlighted by the presence of tall peaks, suggesting that these regions harbour genes or QTLs that strongly influence the traits. The tallest peaks, often far above the threshold, are the most significant, pointing to loci where the genetic variation likely has the greatest impact on the phenotype.

The GWAS analysis conducted on the phenotypic traits of unhulled and hulled rice seeds, specifically targeting size, shape, weight, and color, revealed several significant SNPs across various chromosomes, indicating strong genetic associations. A total of 1,289 associations were identified between SNPs and all phenotypes examined using five GWAS models. Different GWAS models could produce same associations. In this study, of the 1289 associations identified across phenotypes and GWAS models, only 530 non-redundant SNP loci were found to be associated with phenotypes (Supplementary Table 1). For seed size, which includes traits such as area, perimeter, and width, the Manhattan plots indicated significant SNPs predominantly located on chromosomes 1, 3, 5, and 6 (Supplementary Fig. 4a). These chromosomal regions likely harbour genes that influence the overall dimensions of the seed, contributing to variations in seed size. The peaks observed in these regions surpass the genome-wide significance threshold, highlighting the importance of these loci in determining the seed’s physical characteristics. Regarding seed shape, encompassing traits like circularity and roundness, the Manhattan plots also identified significant associations on chromosomes 1, 3, 5, and 6 (Supplementary Fig. 4b). The SNPs located on these chromosomes are likely involved in regulating the curvature and symmetry of the seeds, which are critical factors in defining seed shape. The consistent appearance of significant SNPs on these chromosomes for both seed size and shape suggests that these traits might share some common genetic factors or regulatory pathways. For seed weight, the analysis revealed significant SNPs on chromosomes 3, 5, and 7 (Supplementary Fig. 4c). These chromosomal regions are crucial as they likely contain genes that govern nutrient allocation and the overall seed filling process, which directly impacts the mass of the seeds. The peaks in these regions indicate a strong genetic control over seed weight, with chromosome 5, in particular, showing overlap in significant regions for both size and weight traits, underscoring its central role in seed development. In the case of seed color, significant SNPs were detected on chromosomes 3, 6, and 7 (Supplementary Fig. 4d). These SNPs are associated with genes that influence pigment biosynthesis and deposition within the seed coat, contributing to variations in color traits such as RGB, HSV, Lab, and yCrCb. The identification of these SNPs suggests that multiple chromosomal regions are involved in the regulation of seed color, reflecting the complex genetic architecture underlying this trait. Overall, the GWAS results provide a comprehensive view of the genetic loci associated with key seed traits in rice, with specific chromosomal regions on chromosomes 1, 3, 5, 6, and 7 playing significant roles across different phenotypes.

### Candidate genes for seed related traits in rice

**Fig. 3:**
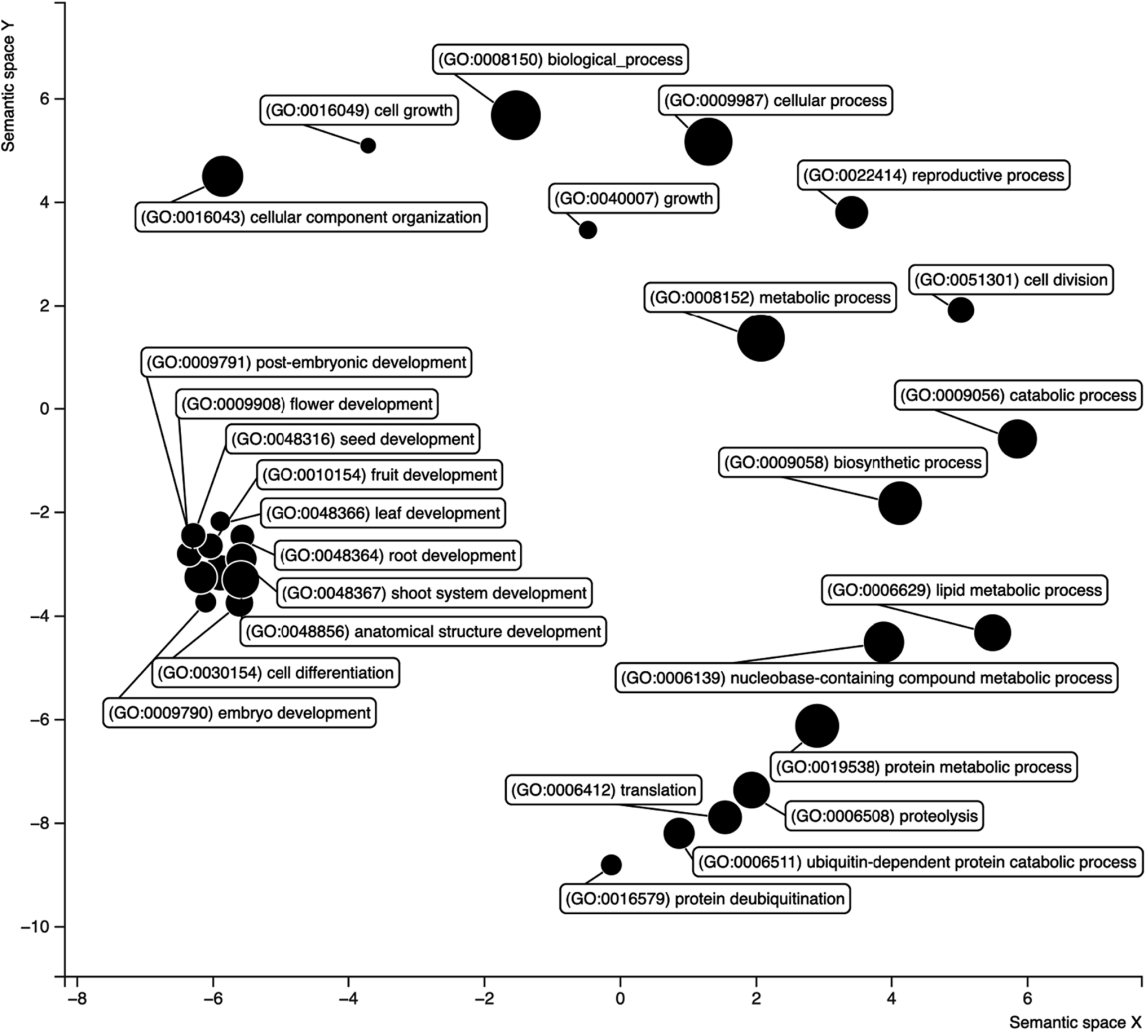
A functional analysis based on gene ontology (GO) was conducted on the biological processes terms of six candidate genes for seed-related traits in rice that were discovered on QTL hotspot rice chromosome 7 and 2. The results were visualised using Revigo software.

The genes within the QTL regions, particularly the QTL hotspots on chromosomes 7 and 2, were extracted, yielding 222 and 104 genes respectively (Supplementary Table 2). Functional analysis was performed on the extracted genes according to gene ontology (GO), yielding 1,143 and 425 terms respectively (Supplementary Table 3). Genes associated with terms associated to seed development were subsequently identified as candidate genes related to seeds in rice plants. Six and seven candidate genes linked to seeds were identified in the QTL hotspot regions of chromosomes 7 and 2 respectively (Table 4). The table highlights candidate genes which are linked to important seed traits such as size, shape, weight, and color. On chromosome 7, Os07g0669800 encodes a cysteine-tryptophan domain-containing zinc finger protein, known as *WG7* or *OsCW-ZF7*, which regulates grain width, influencing seed size and shape. Another gene, Os07g0669500 (*FZP*/*SGDP7*), plays a key role in panicle branching and spikelet formation, directly affecting grain architecture. Similarly, Os07g0671000 encodes *ACL1*, a regulator of internode elongation, which impacts plant height and seed development. Several other genes, such as Os07g0660700 (*OsWD40-149*) and Os07g0658200 (a ribosomal protein gene), contribute to seed development by influencing growth processes. On QTL hotspot chromosome 2, glutelin-related genes such as Os02g0242600 (*GLUBX*), Os02g0248800 (*GLUB6*), Os02g0249000 (*GLUD1*), Os02g0249600 (*GLU7*), Os02g0249800 (*GLUB1A*), and Os02g0249900 (*GLUB1*) were associated with seed storage proteins, which are vital for grain color, filling and weight determination. A gene encoding ubiquitin protease was also identified in this chromosome region that was previously described as a positive regulator of grain width and size in rice. The results of GO analysis, particularly in the category of biological processes, in a total of 13 selected candidate genes showed that many terms were related to developmental processes (Fig. 3). Notably, the analysis identified terms directly related to the traits under investigation, including GO:0009791 (post-embryonic development), GO:0048316 (seed development), GO:0,010,154 (fruit development), GO:009790 (embryo development), and GO:0048856 (anatomical structure development).

**Table 4:** Candidate genes for seed related traits discovered in QTL hotspots on rice chromosome 7 and 2.

Locus ID	Position^†^	Description	Gene name synonym*
Os07g0669800	chr07:28324762..28335077 (+)	Cysteine-tryptophan (CW) domain-containing zinc finger protein, Regulation of grain width (Os07t0669800-01)	*WG7, OsCW-ZF7, CW-ZF7, CWZF7, OsWG7*
Os07g0669500	chr07:28299602..28301089 (−)	ERF transcription factor, Mediation of the transition from spikelet to floret meristem, Determination of panicle branching and spikelet formation (Os07t0669500-01)	*FZP, SGDP7, BFL1, COS1, qSrn7/FZP, qSrn7, fzp, OsBD1, BD1, OsERF#078, OsERF078, OsERF78, ERF78, AP2/EREBP#133, AP2/EREBP133, BFL1, SGDP7, OsSGDP7*
Os07g0675000	chr07:28552641..28558965 (+)	Similar to predicted protein. (Os07t0675000-01); Protein kinase-like domain containing protein. (Os07t0675000-02);Similar to IBR3 (IBA-RESPONSE 3); acyl-CoA dehydrogenase/ oxidoreductase. (Os07t0675000-03)	–
Os07g0658200	chr07:27706359..27709933 (−)	Similar to 30S ribosomal protein S9. (Os07t0658200-01); Similar to 30S ribosomal protein S9. (Os07t0658200-02)	–
Os07g0660700	chr07:27850797..27855081 ( +)	WD repeat protein 55 domain containing protein. (Os07t0660700-01)	*OsWD40-149, OsDWD30, DWD30*
Os07g0671000	chr07:28367865..28368346 (+)	Homologue of Arabidopsis FLOWERING PROMOTING FACTOR1 (FPF1), ACE1 homologue, Control of internode elongation during the reproductive phase (Os07t0671000-01)	*ACL1, FPFL5, OsFPFL5, ACL1, OsACL1*
Os02g0242600	chr02:8057652..8059642 (+)	Similar to Glutelin. (Os02t0242600-01)	*GLUBX, OsGluBX, OsGluBX-J, OsGluBX-I, OsEnS-31, EnS-31, GLU2.1, GluB7, OsGluB7*
Os02g0244300	chr02:8135122..8143345 (+)	Ubiquitin-specific protease, Deubiquitination enzyme, Positive regulation of grain width and size (Os02t0244300-01)	*OsUBP15, LG1, LG1, OsLG1, OsUBP15, UBP15, OsUBP15/LG1, OsUBP7-1, UBP7-1*
Os02g0248800	chr02:8401328..8403216 (−)	Similar to Glutelin type-B 2 precursor. (Os02t0248800-01)	*GLUB6, OsEnS-32, EnS-32, GluB6, OsGluB6*
Os02g0249000	chr02:8406709..8408682 (−)	Glutelin, Seed strage protein (Os02t0249000-01)	*GLUD1, GluD1, GluD-1, OsEnS-33, GLUD1, GluD1, GluD-1, OsGluD-1, OsEnS-33, EnS-33, GluD*
Os02g0249600	chr02:8446623..8448546 (+)	Similar to Glutelin. (Os02t0249600-01)	GLU7, Glu7*(GluB2), Glu7, GluB2, GLUB2, GLUB-2, GluB-2, GluB-7, GLUB7
Os02g0249800	chr02:8458664..8460633 (-)	Glutelin precursor. (Os02t0249800-01)	*GLUB1A, GluB, GluB-1a, OsGluB-1a, GluB-1b*
Os02g0249900	chr02:8465066..8467035 (+)	Glutelin precursor. (Os02t0249900-01)	*GLUB1, GluB1, GluB1-A, GluB-1a, GluB-1, OsGluB1, GluB-1b, OsGluB-1b, GLUB1B*

^†^Position refers to the location of a chromosome, its start and stop points in base pair (bp), and the direction of transcription (positive or negative strand).

*Gene name based on RAP-DB, CGSNL, and Oryzabase gene symbol synonym.

## Discussion

This study comprehensively analyzed the genetic variation in seed size, shape, weight, and color traits in both Indonesian local rice accessions and a recombinant inbred lines (RILs) population through detailed phenotypic assessments and quantitative trait locus (QTL) analysis. The results revealed significant genetic diversity within the local rice accessions compared to the RILs population. Local accessions exhibited broader variability in seed traits than the RILs population (Tables 1 and 2), as evidenced by higher mean values and coefficients of variation for most traits, including seed area, perimeter, and weight. This diversity highlights the rich genetic potential within local varieties, which could be valuable for rice breeding programs aimed at improving specific seed traits. The utilization of these two population types for genetic analysis offers significant advantages, as the strengths of each approach compensate for the weaknesses of the other^12,15,31,32^.

The QTL analysis identified several loci associated with seed size, shape, weight, and color traits, with notable hotspots on chromosomes 7 and 2 (Fig. 1). These hotspots house multiple QTLs, indicating regions with a strong regulatory influence on seed traits (Table 3). For instance, chromosome 7 was identified as a key region, harbouring QTLs that significantly contribute to seed area, length, width, perimeter, shape, and weight, while chromosome 2 harbours QTLs that significantly contribute to seed color. This hotspot underscores the importance of chromosome 7 and 2 in regulating seed morphology and suggests a strong genetic linkage between these traits^33–36^. The presence of multiple QTLs in this region indicates potential pleiotropic effects or tightly linked genes controlling these phenotypes, which could be valuable targets for marker-assisted selection in breeding programs aiming to enhance specific seed characteristics. The study demonstrated that traits such as seed size, shape, and weight are governed by complex genetic interactions, with some traits showing normal distribution patterns, indicative of strong heritability and stable genetic control, while others displayed skewed distributions suggestive of qualitative inheritance influenced by fewer genes (Supplementary Fig. 1). The findings underscore the impact of both genetic and environmental factors on phenotypic expression, particularly in local accessions where broader phenotypic variability was observed (Supplementary Fig. 2). The identification of significant QTLs for seed color across different color spaces (RGB, HSV, Lab, yCbCr) further highlights the complex genetic control of this trait^37–42^. The high LOD scores and explained phenotypic variation for certain color traits suggest strong heritability and a limited influence of environmental factors, making these QTLs reliable markers for color selection in rice breeding. However, the absence of QTLs for some expected traits, such as specific RGB parameters, indicates the potential complexity and polygenic nature of these traits, which may require more refined mapping strategies or larger population sizes to detect. Comparing our findings with existing literature, the genetic control of seed traits observed in this study is consistent with previous reports identifying key loci on chromosomes 3, 4, and 7 related to grain size and shape^40,42–44^. Our results also corroborate earlier findings that highlight the significance of chromosome 7 in controlling seed morphology. Moreover, the identification of QTLs influencing seed color in multiple color spaces aligns with studies that emphasize the polygenic inheritance of color traits, particularly on chromosome 2^45,46^.

The findings from this study align well with existing research on the genetic determinants of seed size, shape, and color in rice, further validating the significance of specific chromosomal regions identified through both GWAS and QTL analysis. The identification of key loci on chromosomes 3, 4, 7, and 2, particularly those related to seed size and weight, is consistent with previous studies. For instance, studies by Xu et al. have reported significant QTLs for grain size and weight on these chromosomes^47^, suggesting that these regions contain critical genes involved in rice seed development. Moreover, the hotspot on chromosome 7 identified in this study, which harbours multiple QTLs influencing seed area, length, and weight, corroborates the findings of Liu et al.^48,49^, who also highlighted the importance of this chromosome in controlling seed morphology. In addition to size and weight, the study’s findings on seed shape traits, particularly the association of circularity and roundness with loci on chromosomes 3, 5, and 7, reflect patterns observed in earlier research. Previous studies also identified similar loci on these chromosome regions that regulate the curvature and symmetry of rice grains^50–53^. These consistent results across multiple studies underscore the robustness of these chromosomal regions in governing key phenotypic traits in rice and suggest that these loci are conserved across different rice populations and environments. Regarding seed color traits, the identification of significant SNPs on chromosomes 2, 3, 6, and 7, especially in the Lab and HSV color spaces, is in agreement with previous research highlighting the genetic complexity of rice seed color. Previous studies also found QTLs for grain color on these chromosomes, particularly noting the involvement of genes related to pigment biosynthesis and deposition^2,37,46,54–56^. The strong association signals observed in this study, particularly on chromosome 7 and 2, suggest that this region plays a crucial role not only in determining seed size and shape but also in controlling seed color, which could have significant implications for rice quality and marketability.

The findings from this research have significant theoretical and practical implications for rice breeding and genetics. The identification of key QTLs and SNPs associated with seed size, shape, weight, and color offers valuable insights into the genetic architecture underlying these complex traits. The discovery of QTL hotspots on chromosomes 7 and 2 (Table 3), including 6 and 7 candidate genes within them (Table 4), which control multiple seed-related traits, underscores the importance of these genomic regions in rice development. Theoretically, these findings contribute to a deeper understanding of the polygenic nature of seed traits in rice, supporting the notion that multiple genes and their interactions are responsible for the expression of these phenotypes. This aligns with previous studies that have demonstrated the polygenic basis of traits such as seed size and weight in rice, where numerous loci across the genome contribute to the overall phenotype^26,57–60^. Practically, the results of this study provide crucial markers for use in marker-assisted selection in rice breeding programs^61,62^. By focusing on the QTLs identified on chromosomes 7 and 2, particularly on the regions flanked by markers S7_27021585 and S7_28324908, as well as S2_7426281 and S2_8559705, breeders can develop rice varieties with optimized seed characteristics, such as improved yield, better grain quality, and enhanced marketability. The high LOD scores (2.3–7.1) and significant phenotypic variation explained by these QTLs (5.33–36.26) indicate that they are robust markers for breeding purposes (Table 3). For example, the identification of QTLs related to seed weight on chromosome 7 offers a clear target for increasing grain yield, a primary objective in rice breeding by modifying grain size and shape^12,53,63^. All QTLs located within the hotspot region on chromosome 7, particularly those related to seed size and weight traits, had negative additive effects, suggesting that the IR64 allele reduces seed area, length, and weight, suggesting that IR64 carries genetic factors that lead to smaller seeds. In contrast, QTLs for shape traits showed positive additive effects, suggesting that alleles from Hawara Bunar contribute to greater circularity and roundness of seeds. Similarly, the QTLs associated with seed color on chromosome 2 could be used to enhance the aesthetic qualities and nutritional value of rice^41,46,64^, which are the important factors for consumer preference. Furthermore, Table 4 highlights candidate genes within QTL hotspots on rice chromosomes 7 and 2, which are linked to seed related traits. On chromosome 7, Os07g0669800 encodes a cysteine-tryptophan domain-containing zinc finger protein, known as *WG7* or *OsCW-ZF7*, which regulates grain width, influencing seed size and shape^65^. Another gene, Os07g0669500 (*FZP*/*SGDP7*), plays a key role in panicle branching and spikelet formation, directly affecting grain architecture^66^. Similarly, Os07g0671000 encodes *ACL1*, a regulator of internode elongation, which impacts plant leaf development^67^. Several other genes, such as Os07g0660700 (*OsWD40-149*) and Os07g0658200 (a ribosomal protein gene), may contribute to seed development by influencing growth processes^68,69^. On chromosome 2, glutelin-related genes such as Os02g0242600 (*GLUBX*), Os02g0244300 (*OsUBP15*), and Os02g0249000 (*GLUD1*) were associated with seed storage proteins, which are vital for grain filling, weight, and nutrient status determination^70^. Seed storage proteins, such as glutelin (the primary storage protein in rice, functionally analogous to glutenin in wheat), play a critical role in determining the end-use quality of rice. Glutelin content affects various aspects of rice quality, including texture, cooking properties, and nutritional value.

These findings provide insights into the genetic mechanisms underlying seed traits and offer valuable targets for breeding programs aimed at improving rice yield and quality through marker-assisted selection. The study also highlights the potential for exploiting genetic diversity in local rice accessions for breeding purposes. The broader genetic variability observed in local accessions compared to the RIL population (Table 1 and 2) suggests that traditional varieties may harbour unique alleles that could be beneficial for breeding programs^71,72^. This aligns with previous research showing that local varieties often possess rare alleles that are lost in modern breeding lines but can be reintroduced to improve traits such as disease resistance, abiotic stress tolerance, and yield. The use of diverse genetic material in breeding programs is crucial for maintaining genetic diversity and ensuring long-term sustainability in rice production.

Despite the significant findings of this study, several limitations should be acknowledged that may affect the interpretation and application of the results. One key limitation is the reliance on a RILs population, which, while beneficial for reducing genetic variability and simplifying the analysis of QTLs, may not fully capture the diversity present in more genetically heterogeneous populations, such as those found in natural or locally adapted rice varieties^15^. This reduced genetic diversity in RILs could potentially lead to the underestimation of QTLs associated with complex traits controlled by multiple loci or gene-environment interactions. Additionally, the use of multiple statistical models in the GWAS increases the robustness of the findings but also introduces complexity in the interpretation of results^30,31^. The variation in results across models, while providing a more comprehensive view, can make it challenging to discern true positive associations from artifacts. This study addressed this issue by focusing on consensus results across models, though discrepancies among models may still contribute to uncertainty in the identified loci. Nevertheless, the limitations of this study can be addressed by combining QTL and GWAS analyses. The linkage mapping summarized the QTL analysis with less complexity (Fig. 1), while GWAS captured a wider genetic variability and detected more loci associated with traits related to grain characteristics (Fig. 2). A region on chromosome 7 was detected to be consistently associated with seed characteristics in both QTL and GWAS analyses.

Building on the findings from this study, future research should focus on fine-mapping the identified QTL to pinpoint specific genes and their regulatory mechanisms underlying seed size, shape, and color in rice. Fine mapping is essential to narrow down the broad QTL regions identified in this study, particularly on chromosomes 7 (S7_27021585 and S7_28324908) and 2 (S2_7426281 and S2_8559705) (Table 3), where multiple traits co-locate, to achieve single-gene resolution. While several genes have been identified as potential candidates linked to seed development regulation (Table 4), over 300 genes within the QTL hotspot region may also play a role in this regulation (Supplementary Table 2), particularly through novel molecular mechanisms that remain unexplored. The candidate genes selected in this study were determined based on functional similarity to genes with established modes of action, as assessed through GO analysis (Fig. 3). This would provide deeper insights into the genetic architecture controlling these traits and facilitate the development of marker-assisted selection strategies for rice breeding programs^62,73^. Advanced techniques such as CRISPR-Cas9 could be employed to validate the function of candidate genes within these QTL regions, confirming their role in trait expression^74^. In addition to fine-mapping, integrating multi-omics approaches, such as transcriptomics, metabolomics, and epigenomics, would be valuable in understanding the gene networks and pathways associated with the identified QTLs^75^. These approaches could reveal how gene expression and metabolite levels influence seed traits under different environmental conditions. For instance, transcriptome analysis across different developmental stages of the seed could identify key regulatory genes and pathways involved in size, shape, and color determination^76,77^. Metabolomics could uncover the biochemical pathways that link gene function to phenotypic traits^78^, while epigenomics could explore the role of DNA methylation and histone modifications in trait variability.

Given the complexity of seed traits, future research should also explore the potential interaction between genetic loci (epistasis) and the environment (G × E interactions). Understanding these interactions is crucial for developing rice varieties that are not only high-yielding but also resilient to varying environmental conditions^79^. Field trials across diverse environments could help identify stable QTLs with minimal environmental influence, ensuring the development of robust rice varieties. Additionally, GWAS in diverse rice world germplasm collections, such as the 3k rice genome project^29^ and the high density rice array^80^ could further enhance the discovery of novel loci associated with seed traits, contributing to broader genetic diversity in breeding programs. Finally, translating these genetic findings into practical breeding applications requires the development of high-throughput phenotyping platforms^81,82^. Such platforms could be used to screen large populations for desirable traits rapidly, integrating phenotypic data with genomic information to accelerate the breeding of improved rice varieties. Collaborative efforts among geneticists, breeders, and computational biologists will be critical in developing these tools and ensuring that the genetic gains achieved in research are efficiently transferred to the field.

## Methods

### Genetic materials

The plant materials used were 90 lines from the Recombinant Inbred Lines (RILs) F_9_ population of rice cv. IR64 and Hawara Bunar, and their parents. This study also used an association panel consisting of 244 local Indonesian rice accessions, part of the 3k rice genome project^29^.

### Seed grain characteristics in RILs population and local rice accessions

The experiment was conducted using a randomized complete block design with three replications. Plants were maintained until they entered the reproductive phase following standard procedures for paddy rice^83^. Uniform-height seedlings were transplanted in an experimental field (altitude: 170 m, coordinates: − 6.56, 106.73) at IPB University, Indonesia. A collection of RIL lines along with their parents and a panel of local rice accessions were cultivated in May 2022. Seedlings for each rice line were transplanted in three rows, comprising 12 plants per row, with a spacing of 30 cm by 15 cm both between and within the rows. Observations were made on panicle architecture, particularly on the traits related to unhulled and hulled seed grain. This stage was performed by generating high-quality digital images by scanning the objects using an Epson Perfection V370 scanner. Scanning was performed by taking grains of each accession as much as 10–15 rice seeds randomly and scanned on a dark background. Calibration of color scale and size was done using X-Rite ColorChecker Classic. The scale used was 0.021 mm per pixel of each image. This step was employed to allow compensation for any color deviations introduced by the camera sensor or lighting conditions during the image processing. The image data of unhulled and hulled grain were deposited on Zenodo (10.5281/zenodo.7699749 and 10.5281/zenodo.7699744).

Digital images obtained from scanning were then measured using SmartGrain software^84^, including area (AS), perimeter length (PL), seed length (L), seed width (W), length-to-width ratio (LWR), and circularity (CS). The loaded image was automatically detected for each trait by counting points sequentially along the seed perimeter. The calculation of AS and PL traits was obtained from the detection of the outer line of the seed while the traits L, W, LWR, and CS were obtained from the detection of the transverse and longitudinal axis lines.

The captured images were first calibrated using the open-source color science Python library Colour. The library automatically detects the presence of the color calibration card and performs color correction on each image. The calibrated images were then processed using the OpenCV Python image processing library^85^. In each image, the seeds were segmented using the findContours function in OpenCV. This function identifies continuous edges in the image and finds contours that can be isolated from the background. Each segmented contour represents individual seeds in the background. For each segmented seed, color features were extracted in four different color spaces: Red–Green–Blue (RGB), Hue-Saturation-Value (HSV), Lab (L*a*b*), and YCbCr. These color spaces were chosen for their ability to represent color in ways that align with human visual perception and digital color processing needs. The mean and standard deviation of the color channels in each color space were computed for every seed contour, capturing both the central tendency and variability of colors within each seed. The process resulted in a total of 16 color features per seed. These features serve as the foundation for differentiating between seed specimens based on their color characteristics.

### QTL mapping

The average trait value of plants from each RIL was used for QTL analysis. QTL analysis was performed using the Inclusive Composite Interval Mapping based on phenotypic data of all seed grain-related traits and a genotype matrix along with the 1,980 cM linkage map that was constructed from SNP markers generated by IR64 × Hawara Bunar RIL and their parental genome sequences in our previous study^8^. A total of 873 bin markers representing a segregation of 55,205 high quality SNP markers from the RIL population were used as genotypes to conduct QTL analysis. The SNP markers were previously generated from the filtering step with the criteria of a minimum minor allele frequency of 10%, a minimum genotype call of 90%, and a depth of coverage of at least 10x. The logarithm of odds (LOD) threshold value is set at 2.5 to determine the significance of the QTL existence in the QTL.gCIMapping package in R software^86^. Since the average length of the 12 rice chromosomes on the linkage map constructed in our previous research was approximately 150 cM, a LOD value of 2.5 is sufficient to justify approximately 98% probability of a significant QTL in this study^87^.

### Population structure and association analyses

The resequencing data for 244 rice accessions were released in NCBI, and information on variation sites is provided in the SNP-Seek database (https://snp-seek.irri.org). We used PLINK^88^ with -maf 0.05, -geno 0.1, and -hwe 1e−10 to screen 1,011,601 SNPs and found 334,776 with minor allele frequency more than 5%. The population structure (PCA and kinship matrices) and the genome-wide association (GWAS) were computed using the R package Genomic Association and Prediction Integrated Tool (GAPIT) with default parameters^89^. Five statistical models (GLM, MLM, farmCPU, BLINK, and SUPER) were employed to discover associations between SNPs and traits of interest. The MLM utilizes both PCA and kinship, with PCA adjusting for population structure and the kinship matrix modelling random effects due to relatedness. The farmCPU model integrates kinship and principal components to control for both fixed and random effects. The BLINK model incorporates kinship and principal components, improving the detection of significant loci by controlling for population structure and relatedness. The SUPER model uses kinship to account for genetic relatedness, enhancing the power to detect true associations^17^. All probabilities in association runs were transformed by − log10 *P* (FDR *p*-value < 0.05). Scores for each chromosome were then examined in Manhattan plots to evaluate whether the SNPs met the significance level. Multiple test correction was used to alter the − log10 *p*-values of SNPs from GWAS. The corrected cutoff value for the accepted thresholds was − log10 (*P*) value ≥ 7.0.

### Identification of candidate genes for seed related traits

Genes within the QTL hotspot regions, particularly on chromosome 7 and 2 flanked by the markers S7_27021585 and S7_28324908, as well as S2_7426281 and S2_8559705, were extracted using BioMart software^90^ from Plant Ensembl (https://plants.ensembl.org/biomart) on *Oryza sativa* Japonica Group genes (IRGSP-1.0). Gene ontology (GO) attributes were extracted for each selected gene, which were then manually curated based on keywords containing the term “development” or “seed”, to select candidate genes related to developmental processes in seed grain. All GO terms in the selected candidate genes were visualized using Revigo software^91^. The descriptions and name of the selected candidate genes were searched on the rice annotation project database (RAP-DB, https://rapdb.dna.affrc.go.jp)^92^.

## Supplementary Information

Supplementary Information 1.

Supplementary Information 2.

## Data Availability

Data are provided within the manuscript or supplementary information files. The image data of seed grain generated in this study were deposited on Zenodo (https://doi.org/10.5281/zenodo.7699749 and https://doi.org/10.5281/zenodo.7699744).
