# Supplementary material for: Rice QTL hotspots related with seed grain size, shape, weight, and color based on genome wide association study and linkage mapping
Source: Sci Rep. 2025 Jul 1;15:21470. doi: 10.1038/s41598-025-05814-3 (PMC12218392; doi:10.1038/s41598-025-05814-3)
Supplement: Supplementary file 1 — Supplementary Information 1. [file 41598_2025_5814_MOESM1_ESM.pdf]

Supplementary Fig. 1. Phenotypic distribution of seed grain characteristics in local rice accessions.

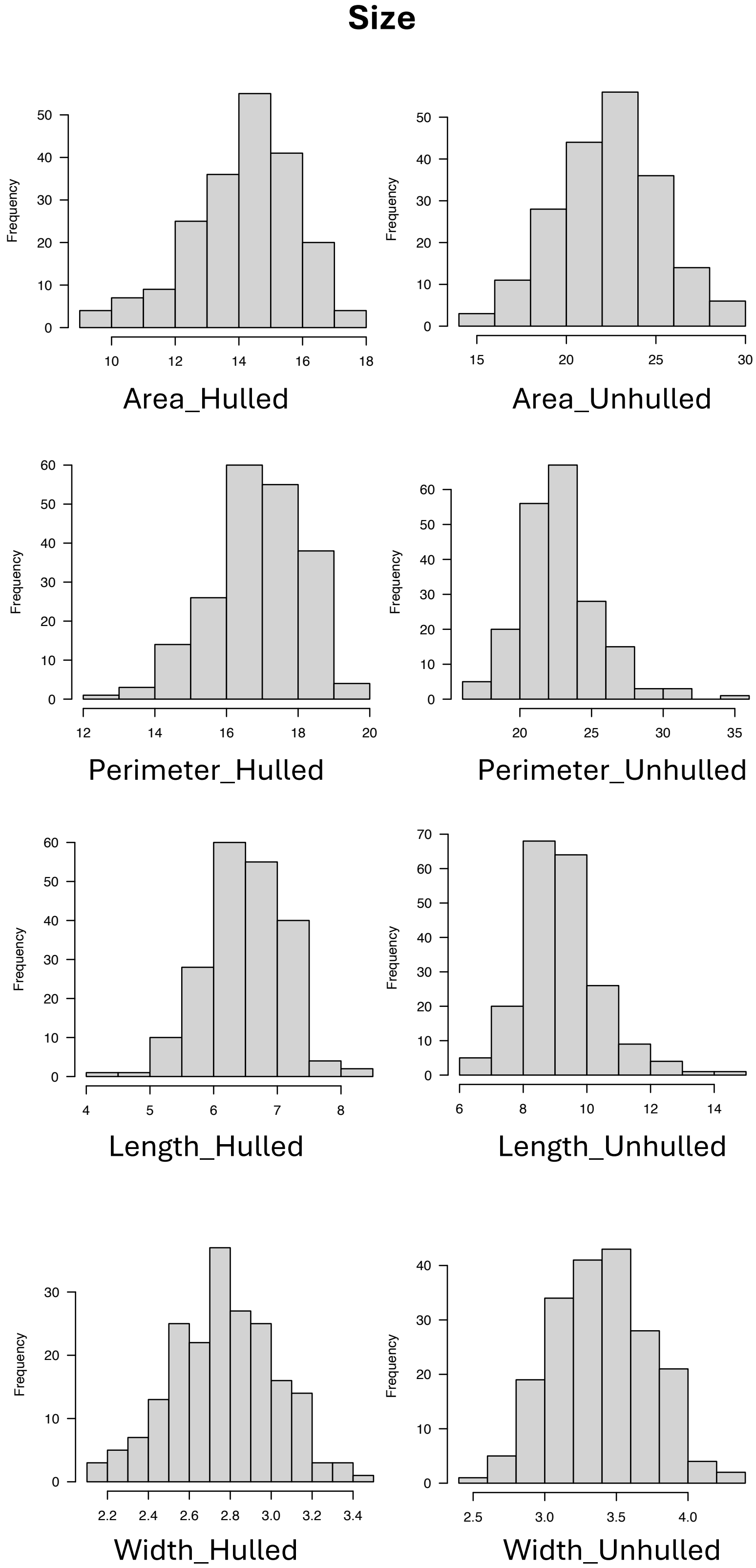

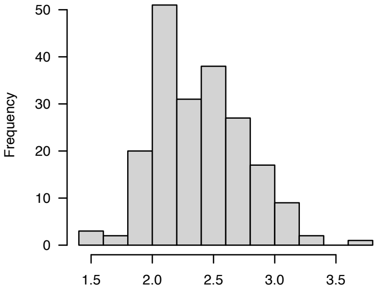

LtW_Hulled

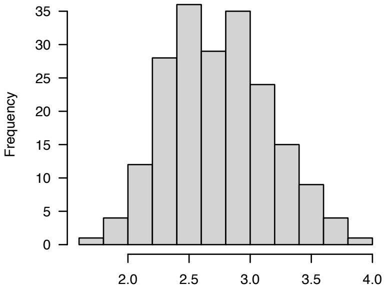

LtW_Unhulled

Shape

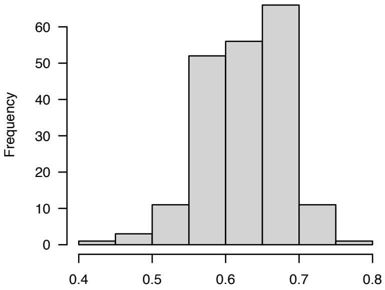

Circularity_Hulled

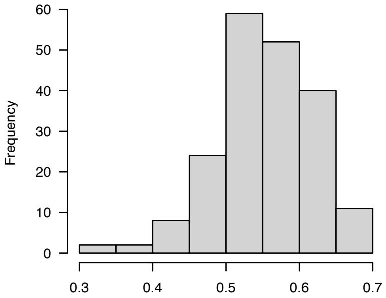

Circularity_Unhulled

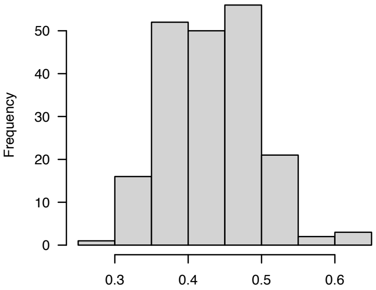

Roundness_Hulled

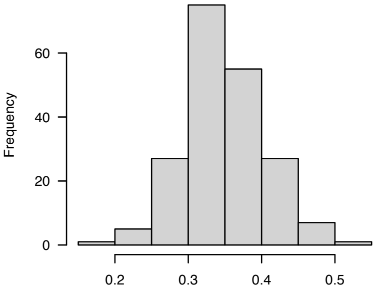

Roundness_Unhulled

Weight

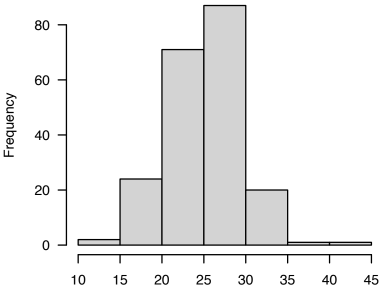

Seed_1000

Color

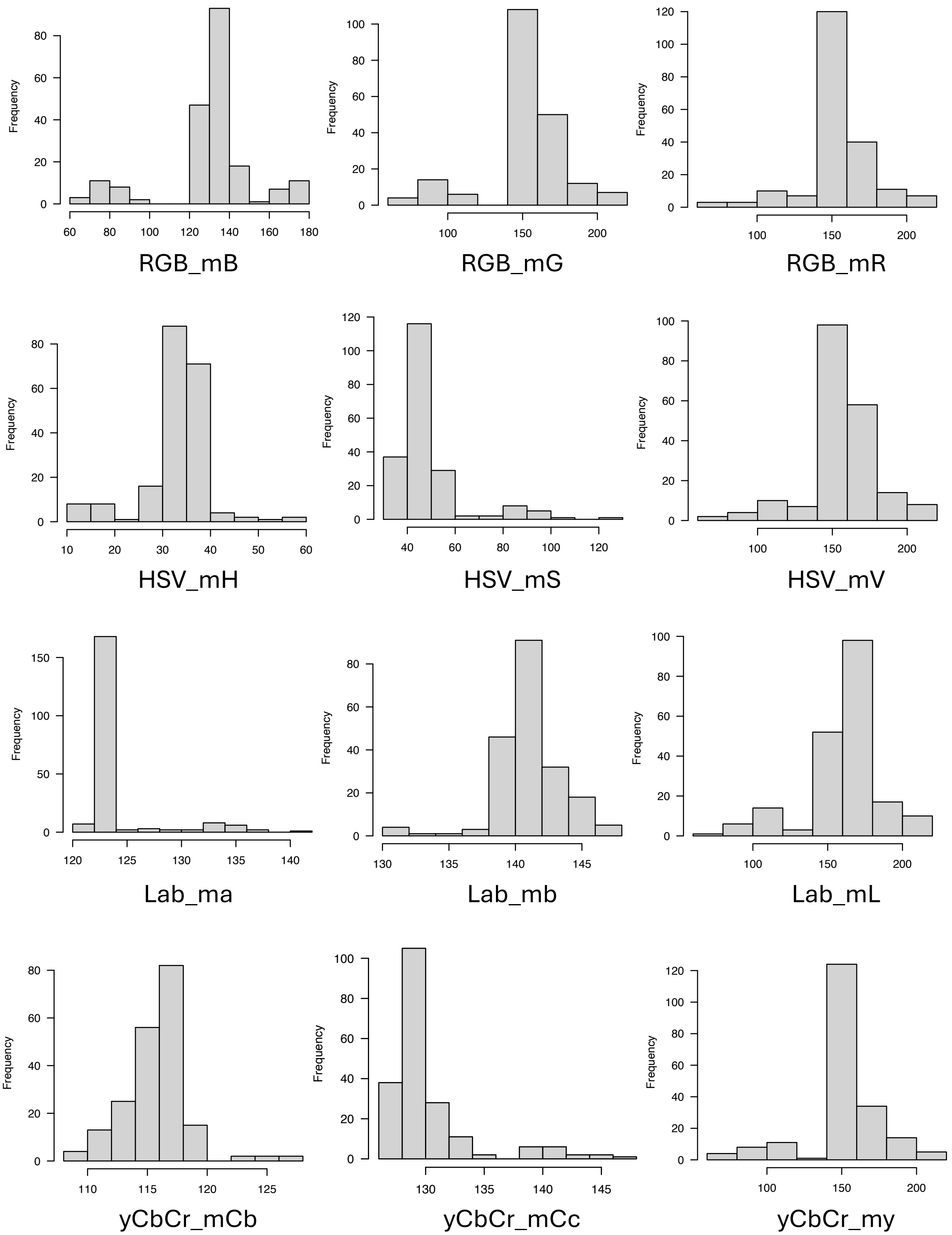
Frequency
RGB_mB
RGB_mG
RGB_mR
HSV_mH
HSV_mS
HSV_mV
Lab_ma
Lab_mb
Lab_mL
yCbCr_mCb
yCbCr_mCc
yCbCr_my

Supplementary Fig. 2. Phenotypic distribution of seed grain characteristics in recombinant inbred lines (RILs) population. Red and blue arrow indicated the Hawara Bunar and IR64, respectively, as the parental lines of RILs.

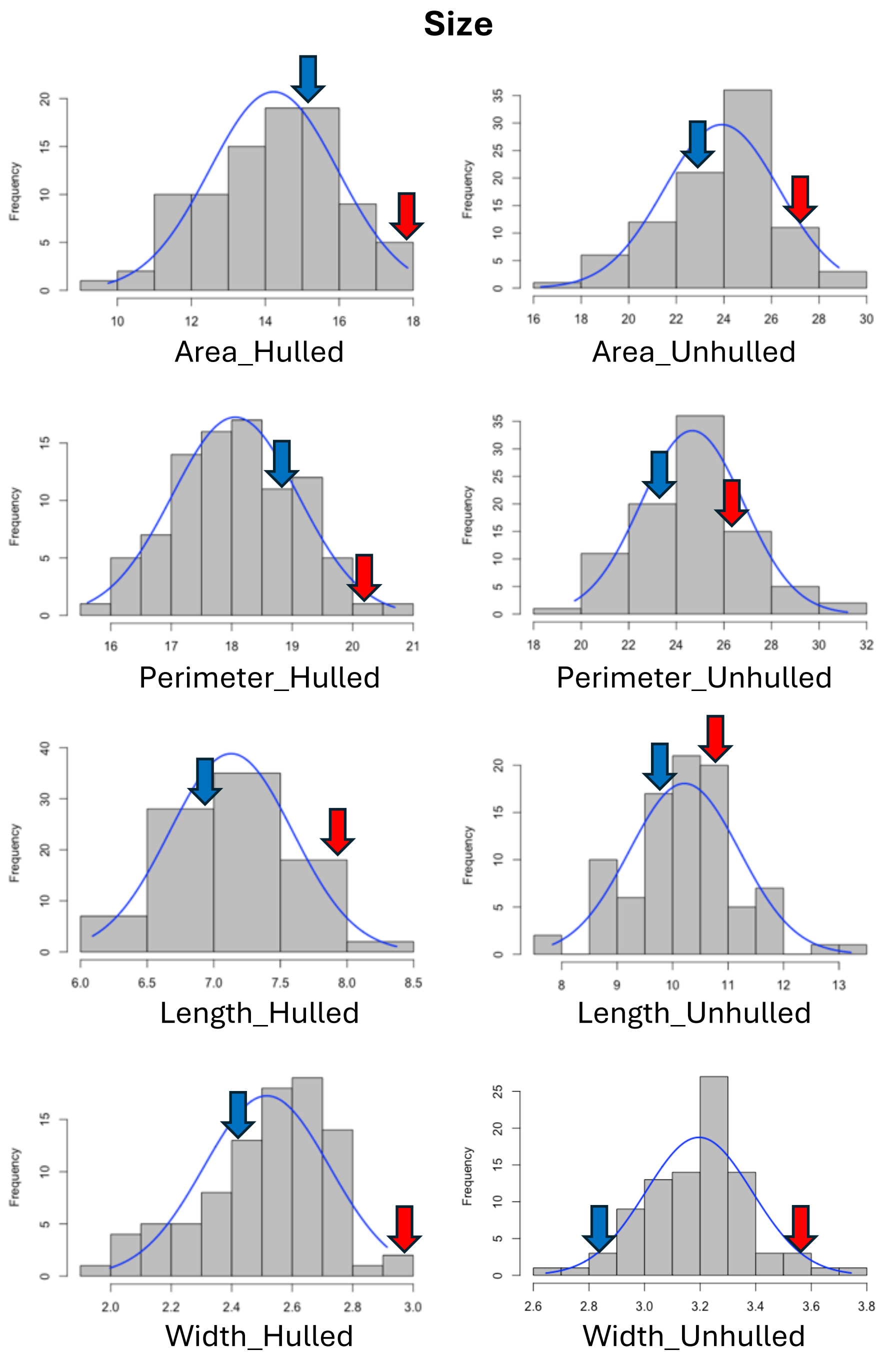

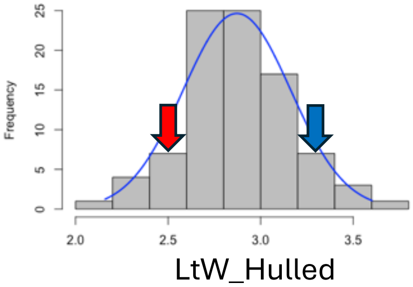

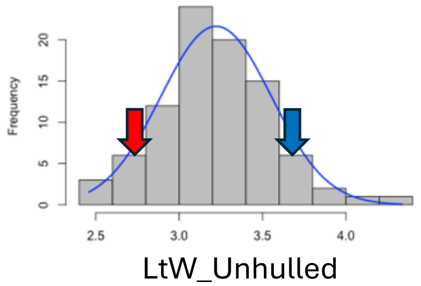

Shape

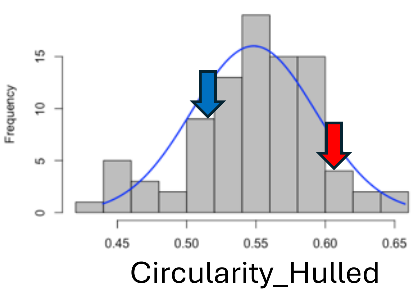

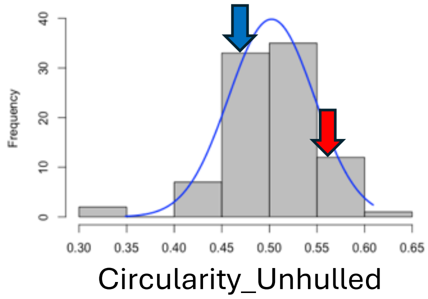

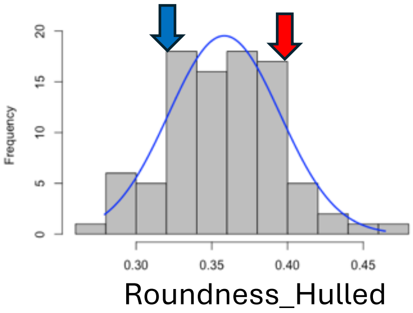

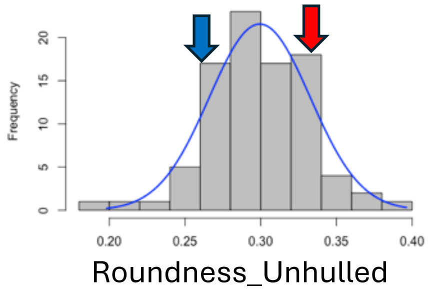

Weight

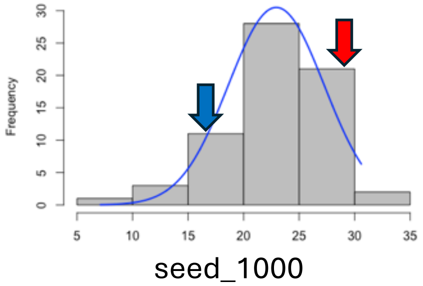

Color

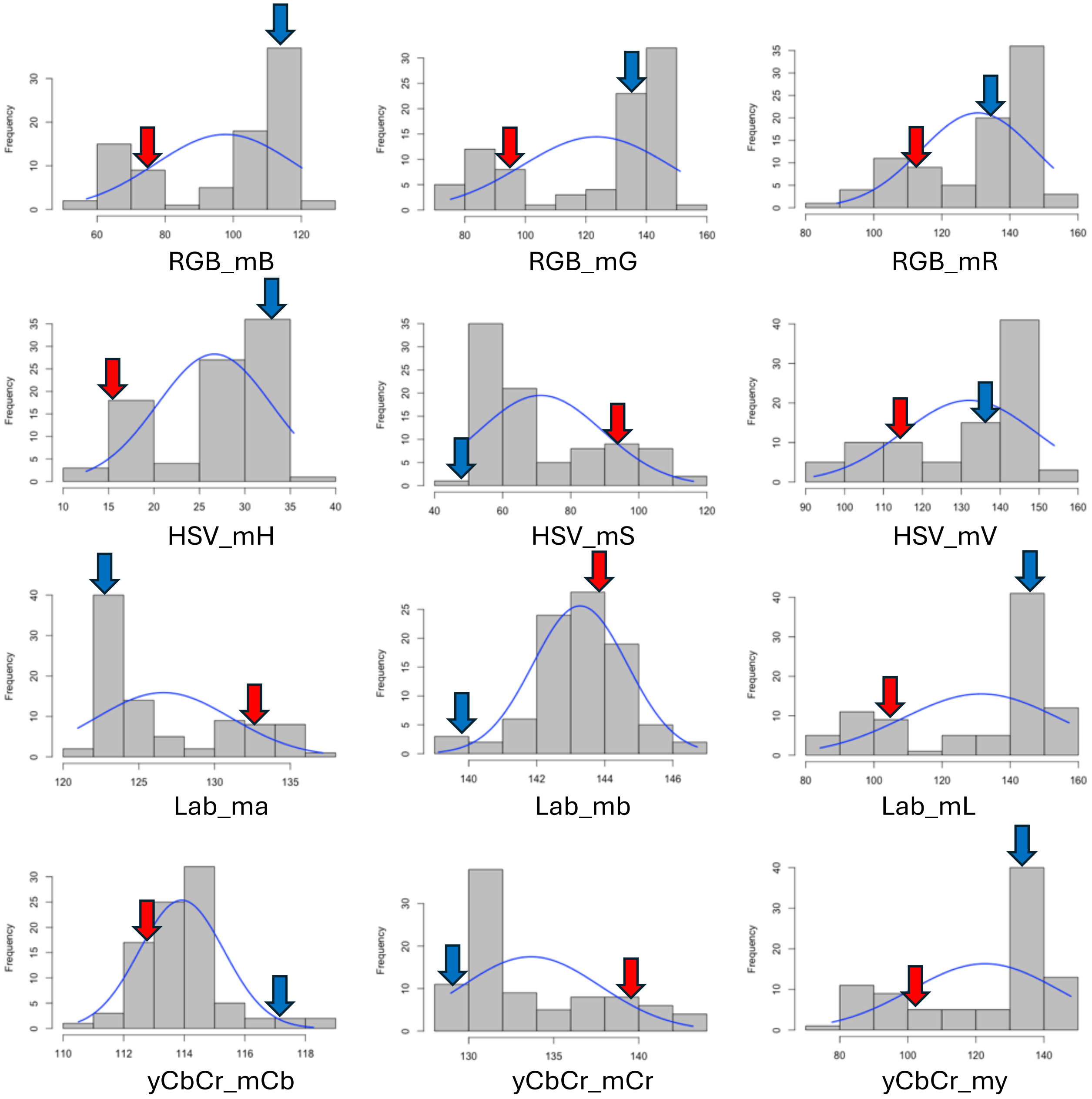

Frequency
RGB_mB
RGB_mG
RGB_mR
HSV_mH
HSV_mS
HSV_mV
Lab_ma
Lab_mb
Lab_mL
yCbCr_mCb
yCbCr_mCr
yCbCr_my

Supplementary Fig. 3. Population structure represented by principal component analysis (PCA) and kinship. (a) PCA plot, (b) eigen value plot, (c) kinship plot.

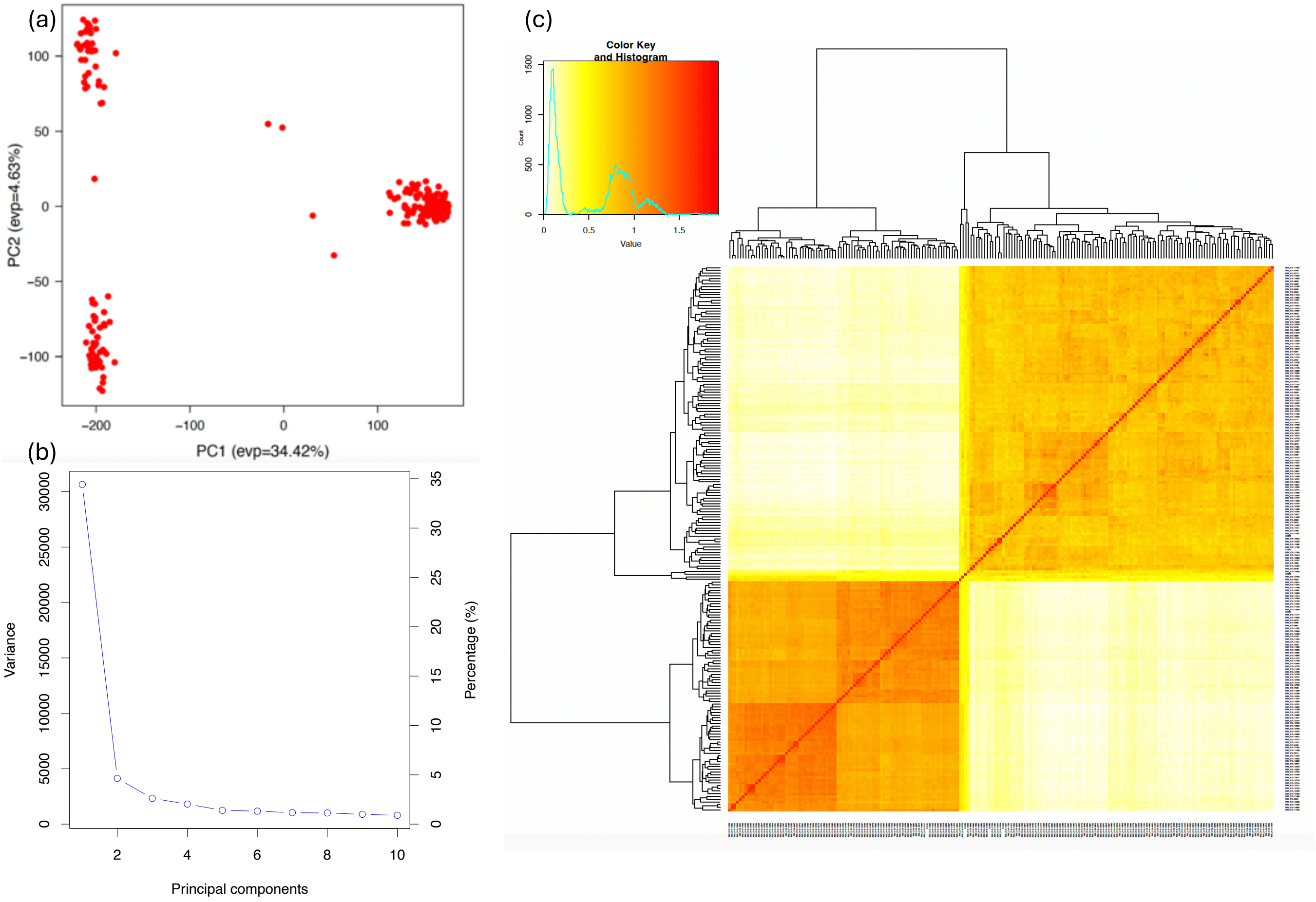

Supplementary Fig. 4. Manhattan plot for seed characteristics detected in local rice accessions. GWAS analyzed in seed size (a), shape (b), weight (c), and color (d).

(a)

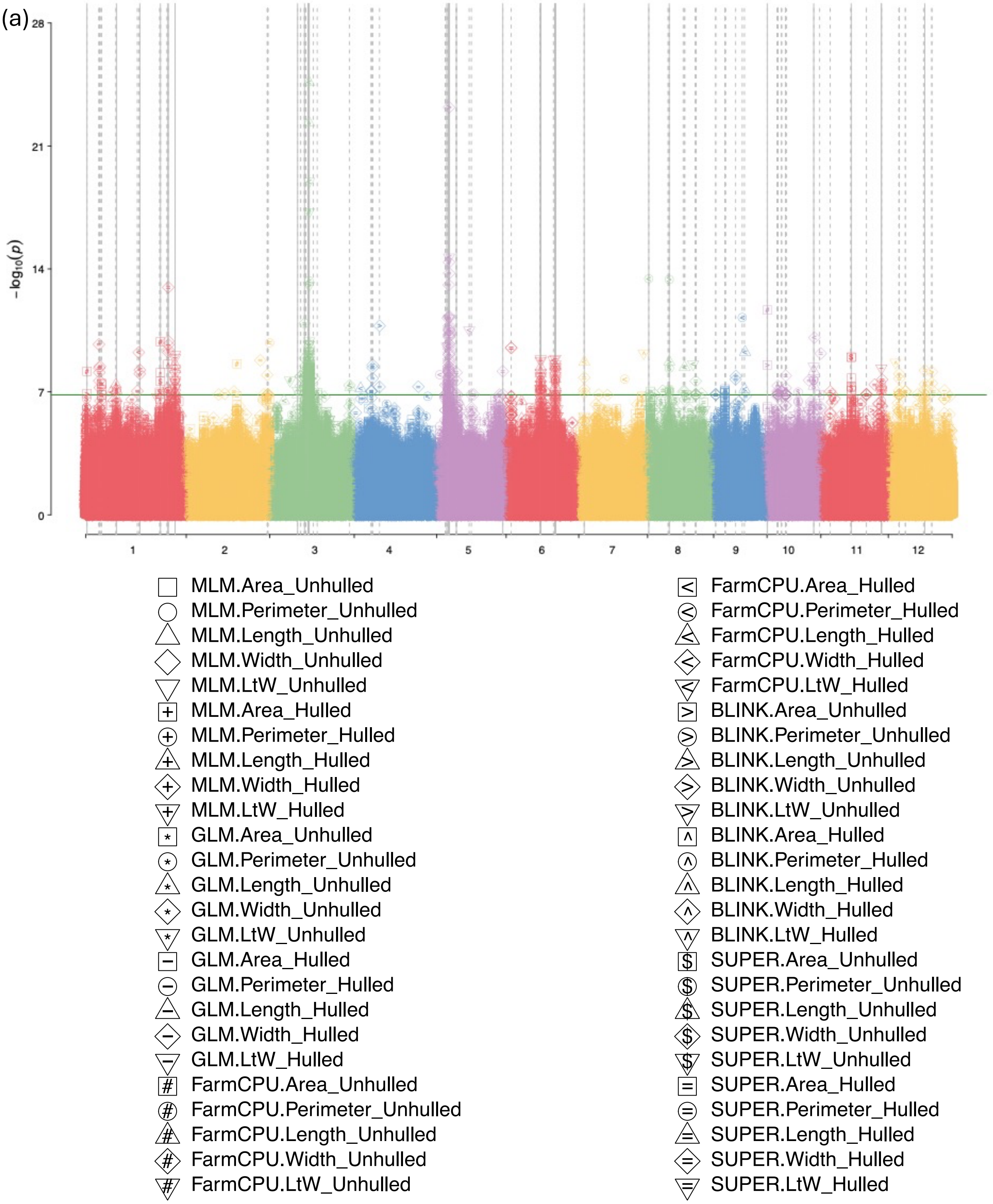

Manhattan plot fot seed **size**, including area, perimeter, length, width, and length-to-width ratio

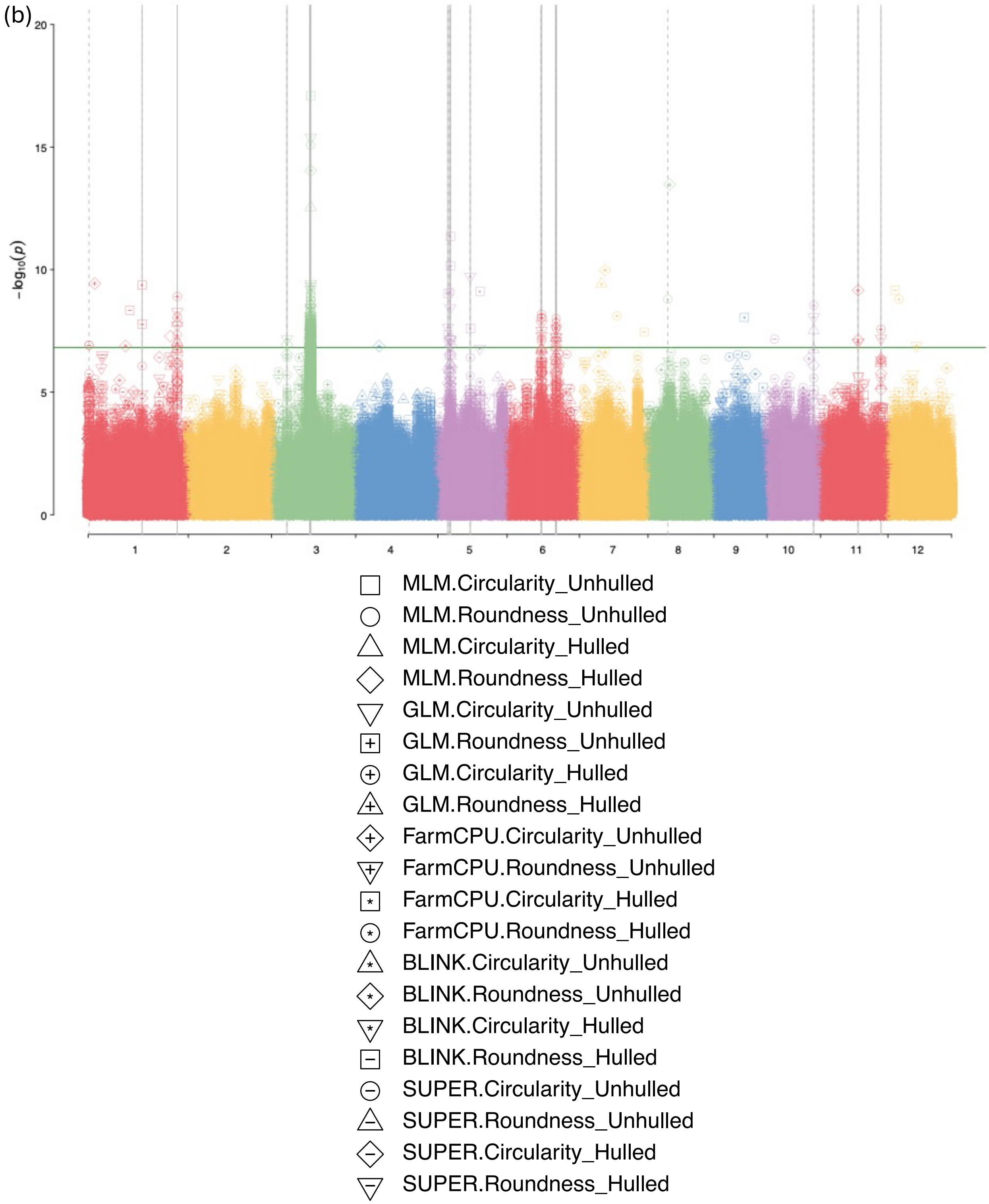

Manhattan plot for seed **shape**, including circularity and roundness

(c)

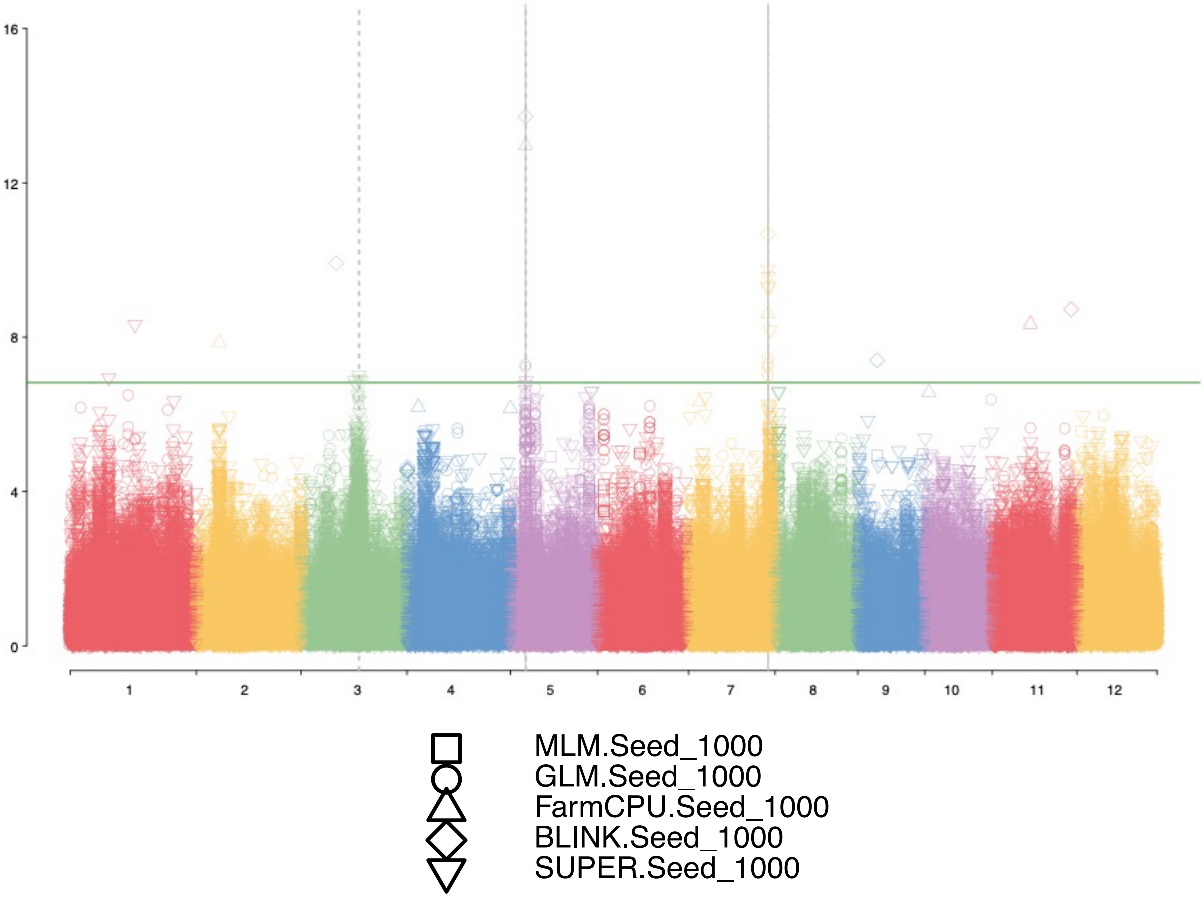

Manhattan plot for seed **weight**

(d)

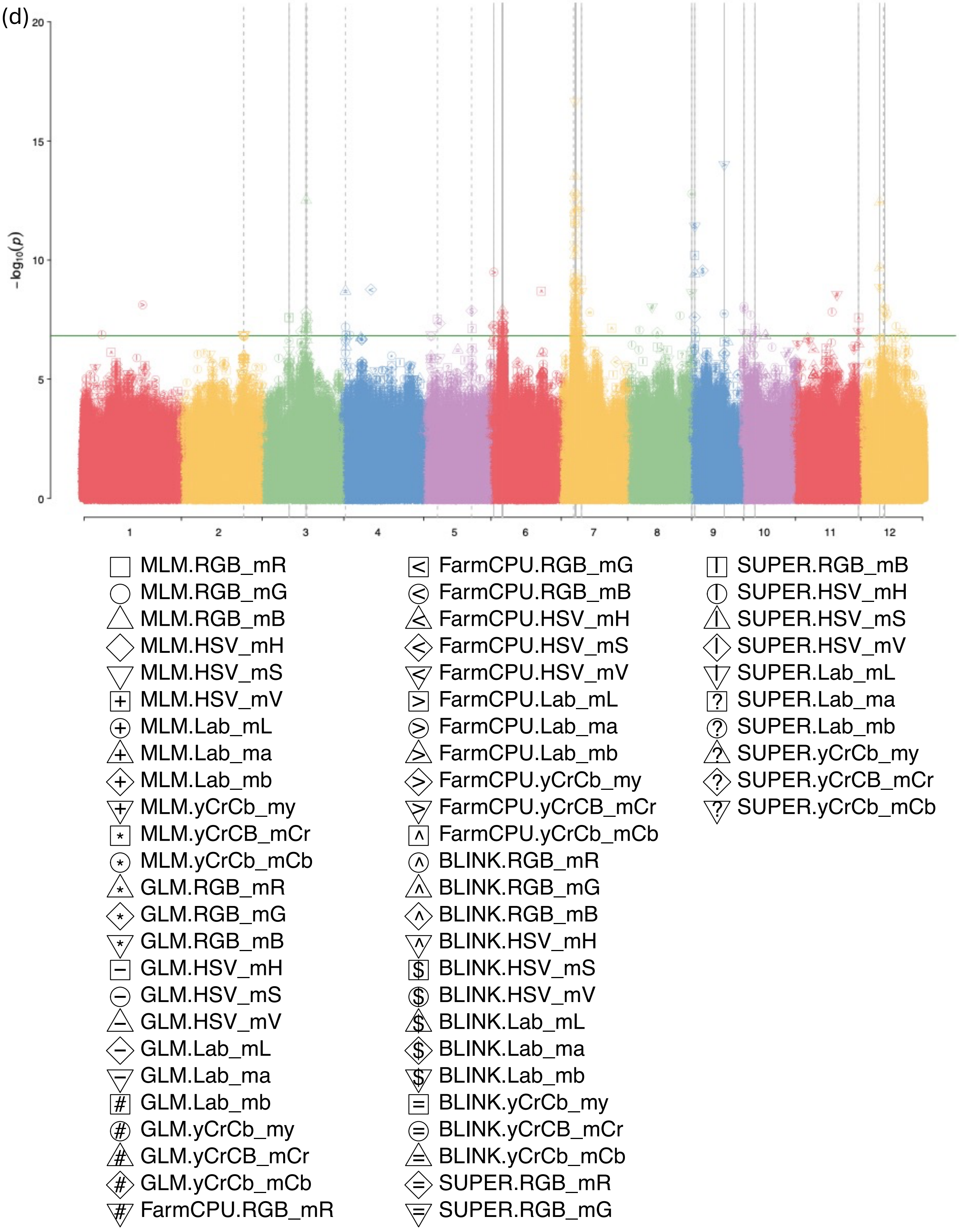

Manhattan plot for seed **color**, based on RGB, HSV, Lab, and yCbCr system
